# Interactions of *N*-Mannich Bases of Pyrrolo[3,4-*c*]pyrrole with Artificial Models of Cell Membranes and Plasma Proteins, Evaluation of Anti-Inflammatory and Antioxidant Activity

**DOI:** 10.3390/membranes13030349

**Published:** 2023-03-17

**Authors:** Łukasz Szczukowski, Jadwiga Maniewska, Benita Wiatrak, Paulina Jawień, Edward Krzyżak, Aleksandra Kotynia, Aleksandra Marciniak, Maciej Janeczek, Aleksandra Redzicka

**Affiliations:** 1 Department of Medicinal Chemistry, Faculty of Pharmacy, Wroclaw Medical University, Borowska 211, 50-556 Wroclaw, Poland; 2 Department of Pharmacology, Faculty of Medicine, Wroclaw Medical University, Mikulicza-Radeckiego 2, 50-345 Wroclaw, Poland; 3 Department of Biostructure and Animal Physiology, Division of Animal Anatomy, Faculty of Veterinary Medicine, Wroclaw University of Environmental and Life Sciences, Kożuchowska 1, 51-631 Wroclaw, Poland; 4 Department of Basic Chemical Sciences, Faculty of Pharmacy, Wroclaw Medical University, Borowska 211a, 50-556 Wroclaw, Poland

**Keywords:** ADME, drug–membrane interaction, DSC, fluorescence spectroscopy, HSA, inflammation, lipoxygenase, molecular docking study, oxidative stress

## Abstract

Despite the widespread and easy access to NSAIDs, effective and safe treatment of various inflammatory disorders is still a serious challenge because of the severe adverse effects distinctive to these drugs. The Mannich base derivatives of pyrrolo[3,4-*c*]pyrrole are potent, preferential COX-2 inhibitors with a COX-2/COX-1 inhibitory ratio better than meloxicam. Therefore, we chose the six most promising molecules and subjected them to further in-depth research. The current study presents the extensive biological, spectroscopic and in silico evaluation of the activity and physicochemical properties of pyrrolo[3,4-*c*]pyrrole derivatives. Aware of the advantages of dual COX–LOX inhibition, we investigated the 15-LOX inhibitory activity of these molecules. We also examined their antioxidant effect in several in vitro experiments in a protection and regeneration model. Furthermore, we defined how studied compounds interact with artificial models of cell membranes, which is extremely important for drugs administered orally with an intracellular target. The interactions and binding mode of the derivatives with the most abundant plasma proteins—human serum albumin and alpha-1-acid glycoprotein—are also described. Finally, we used computational techniques to evaluate their pharmacokinetic properties. According to the obtained results, we can state that pyrrolo[3,4-*c*]pyrrole derivatives are promising anti-inflammatory and antioxidant agents with potentially good membrane permeability.

## 1. Introduction

Semipermeable plasma membrane surrounds the cytoplasm of all living cells, thereby separating the intracellular and the extracellular environment and playing a crucial role in maintaining the biological and chemical differences between those areas [1,2]. Although the cells were first described by the eminent scientist Robert Hooke in about 1665, it was not until the mid-19th century that the existence of the cell membrane was discovered, alongside the development and formulation of cell theory [2]. Our understanding and knowledge of biological membranes have been gradually and considerably enhanced with time. The cell membranes are commonly considered highly complex, supramolecular liquid-crystalline structures built on the phospholipid bilayer framework linked by proteins and carbohydrates. So far, the liquid mosaic model proposed by Singer and Nicolson in 1972, although simplified indeed, describes as well as possible the structure, organization, and, significantly, the dynamic properties of biological membranes [1,2,3,4,5].

Nevertheless, the cell membrane not only acts as an ordinary, simple barrier. It protects the cell against harmful external stimuli and, above that, is engaged in a great variety of biochemical, biosynthetic, signaling, sorting, metabolic and many other processes, which are essential for the proper functioning of the cell, tissue, and as a result, the whole organism. The vast majority of cellular functions occur in the plasma membrane or its proximity, which explains its extreme importance in the living organism [1,2,3,5,6].

It is worth noting that phospholipids not only provide the scaffolding of the cell membrane but also play a significant role in the initiation, development and course of multiple physiological and pathological processes. The hydrolysis of membrane glycerophospholipids catalyzed by cytosolic phospholipase A_2_ (cPLA_2_) results in the release of polyunsaturated fatty acids (PUFAs), mainly arachidonic acid (AA) [1,2,3,6,7]. It is the first step of the biosynthesis of a broad group of compounds named eicosanoids, which represent extensively studied and probably the best-characterized type of bioactive lipids [6,8,9]. When AA enters the cytosol, it undergoes a multistep transformation conducted by three different enzymatic pathways. The first depends on cyclooxygenases (COXs) which exist mainly peripherally in two isoforms—constitutive COX-1 and induced COX-2. These membrane-bound enzymes are found on the internal and external nuclear envelope membranes and the luminal surface of the endoplasmic reticulum (ER). COXs are responsible for dioxygenation followed by the reduction of AA. This leads to the formation of prostaglandin H_2_ (PGH_2_), which can be subsequently turned into a wide range of prostanoids—prostaglandins (PGs) and thromboxanes (TXs) [3,6,7,8,9,10,11,12,13]. On the other hand, when AA becomes a substrate for lipoxygenases (LOXs), which constitute a family of non-heme iron-containing enzymes, polyenes such as leukotrienes (LTs) and lipoxins are formed. When considering the LOX pathway, the three isoforms of the greatest importance are 5-, 12- and 15-LOX. These enzymes take their names from the number of the AA carbon atom, the oxidation of which they catalyze. Finally, cytochrome P450 epoxygenases are engaged in the biosynthesis of epoxide and hydroxide derivatives of AA [7,14,15,16].

Beyond any doubt, prostanoids are the most abundant class of eicosanoids [7]. These autacoids are engaged in many physiological functions. They exert a protective effect on the gastric and duodenal mucosa by increasing the production of mucus and bicarbonates. PGs, especially PGI_2_, reduce blood coagulability and cause vasodilatation. PGs are also very important inflammatory mediators, whose expression significantly raises in inflamed tissue [1,3,7,11,12]. They are responsible for the initiation and development of inflammation and pain and can also affect the expression of other mediators. Lipoxins and LTs also play a crucial role in the inflammatory response: they promote leukocyte chemotaxis and recruit and activate various types of T cells, among others CD8+ “killer” T cells. Therefore, this group of eicosanoids is involved in both the induction and resolution of inflammation. Overexpression of 15-LOX is observed in many inflammatory disorders, such as asthma or osteoarthritis [6,7,8,9,14,15].

Both isoforms of COX are molecular targets for nonsteroidal and anti-inflammatory drugs (NSAIDs) [11,12,17]. The great majority of these medicaments are nonselective inhibitors of these enzymes. As a consequence, the biosynthesis of prostanoids decreases, which leads to the relief of pain and inflammation. Unfortunately, the reduced quantity of PGs is the direct cause of the characteristic adverse effects of NSAIDs, mainly related to the gastrointestinal tract (GIT), such as heartburn, bleeding or ulceration. Although selective COX-2 inhibitors (COXIBs) generally spare GIT, they can cause serious cardiovascular (CV) side effects with thromboembolic events, which may result in myocardial infarction or stroke [18,19,20,21,22,23,24]. Adverse effects of NSAIDs are also associated with their influence on cell membranes and impairment of the function of surface-active phospholipids. Moreover, long-term inhibition of COX accompanying therapy with NSAIDs may shift the metabolism of AA into the LOX pathway. This can cause bronchoconstriction and airway inflammation, provoked by the higher level of LTs. Taking all this into account, effective and safe therapy, especially for chronic inflammatory disorders, is still limited by the severe adverse effects of commonly available drugs. Therefore, searching for new, potent, secure analgesic and anti-inflammatory agents is still challenging. The development of dual COX–LOX inhibitors might be a significant step towards the efficient management of inflammation [1,6,7,8,10,11,12,13,14,15,16].

In our previous paper, we reported the synthesis and biological evaluation of a series of Mannich base derivatives of pyrrolo[3,4-*c*]pyrrole. These compounds showed promising anti-COX activity. On enzymatic assay, all investigated molecules inhibited an inducible isoform COX-2 stronger than COX-1. Moreover, every studied compound revealed a lower IC_50_ value of COX-2 inhibition and a better COX-2/COX-1 selectivity ratio than the reference drug—meloxicam. These findings were supported by molecular docking studies, which showed that pyrrolo[3,4-*c*]pyrrole Mannich bases take a position in the active site of COX very similar to meloxicam [25].

**Figure 1:**
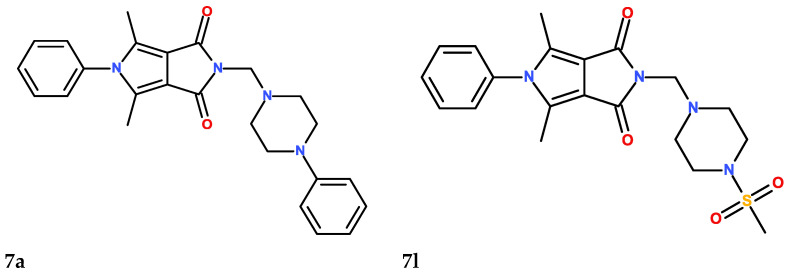

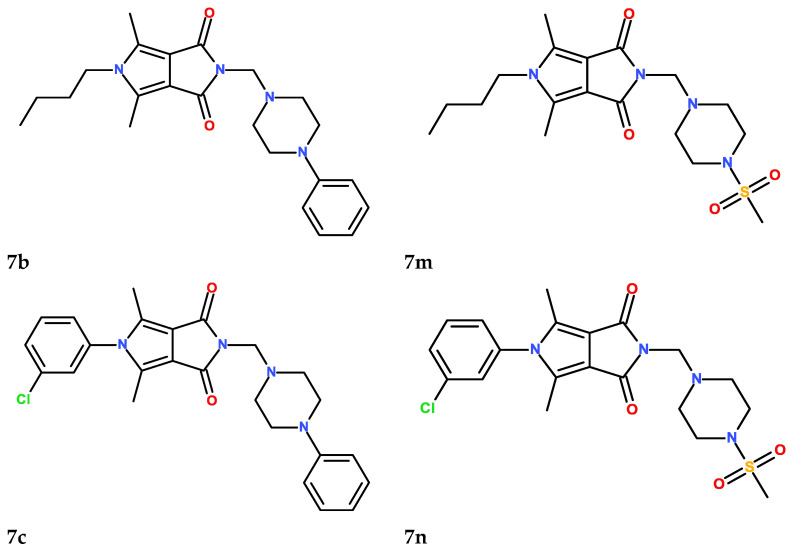
The structures of the six (**7a**–**7c**, **7l**–**7n**) investigated *N*-Mannich base derivatives of pyrrolo[3,4-*c*]pyrrole.

Inspired by those results, we decided to choose the six most promising compounds (Figure 1) and perform further, more advanced experiments to determine the activity, mechanism of action and pharmacokinetic properties of the pyrrolo[3,4-*c*]pyrrole Mannich base derivatives. Based on the structure of the investigated molecules, they have different aliphatic or aromatic substituents in position C5 of the pyrrolo[3,4-*c*]pyrrole. Additionally, in the structure of compounds **7a**–**7c**, a phenylpiperazine moiety can be distinguished, while derivatives **7l**–**7n** possess the methylsulfonylpiperazine pharmacophore (Figure 1). Aware of the advantages of dual COX–LOX inhibition [14,15,26,27,28,29], we determined the inhibition activity of the compounds towards the 15-LOX enzyme. The inhibition potency, the binding mode and the affinity to the active site of 15-LOX were defined in vitro using an enzymatic kit and molecular docking studies. Moreover, we analyzed the interaction of the compounds with artificial models of biological membranes. Such experiments are crucial in the case of molecules that are administrated orally and have an intracellular target. Drug–membrane interaction studies help to understand the pharmacokinetics, mechanism of action and toxicity of investigated structures [1,2,3,5,7]. Taking into consideration that inflammation is usually accompanied by oxidative stress, which can cause lipid peroxidation and cell damage, we also determined the antioxidant activity of pyrrolo[3,4-*c*]pyrrole derivatives and their protective role against reactive oxygen and nitrogen species (ROS and RNS) [6,9,11,16,30,31,32,33]. The interactions of the compounds with the most abundant plasma proteins were described by spectroscopic and molecular docking studies [34,35]. Finally, we estimated some physicochemical properties of subjected Mannich bases using in silico techniques [36,37]. We performed all experiments to characterize the best-possible biological, physical and chemical features of Mannich base derivatives of pyrrolo[3,4-*c*]pyrrole, a potentially new, effective class of anti-inflammatory agents.

## 2. Materials and Methods

### 2.1. Biological Evaluation

#### 2.1.1. Cell Line and Culture Conditions

Normal human dermal fibroblast (NHDF) obtained from the American Type Culture Collection (ATCC) were cultured in a medium recommended for this cell line—DMEM with 4.5 g/mL glucose without phenol red—which was supplemented with 10% FBS and 2 mM L-glutamine and 25 μg/mL gentamicin. The cells were incubated in 5% CO_2_, 37 °C, and 95% humidity and were evaluated twice a week. If the confluence was above 70%, the cells were subcultured with TrypLE solution and used in biological assays or reduced. In all studies, cells were seeded at 10,000 cells/well density and incubated for 24 h to regenerate in a CO_2_ incubator.

#### 2.1.2. Tested Compounds

Tested compounds were dissolved in DMSO to a final concentration of 10 μM and stored at −20 °C. Before use, the compound solution was kept at room temperature until thawing, and then samples with 3 different concentrations were prepared (100 µM, 50 µM and 10 µM) for each compound. The tested concentrations of compounds were prepared in a culture medium. At the highest concentration of each compound, the DMSO content did not exceed 1%.

#### 2.1.3. Lipoxygenase Inhibition Assay

Cayman’s lipoxygenase inhibitor screening assay kit (760700) (Cayman Chemical, 1180 East Ellsworth Road, Ann Arbor, MI 48108, USA) was used according to the manufacturer’s instructions. The test detected and measured hydroperoxides produced in the lipoxygenation reaction using purified LO. All details concerning the conducted experiment are described in the instructions provided by the supplier. Readings were performed using a VarioScan Go plate reader at a wavelength of 490 nm.

#### 2.1.4. Experimental Design

All studies included the treatment of cells in two different ways, as follows.

1. Protective properties against ROS or NO (RNS) formation

After 24 h of cell regeneration, the medium was removed and solutions of the tested compounds (each at three concentrations: 10, 50 and 100 μg/mL) were added for a further 24 h. After this time, the solutions were removed. Next, the cells were exposed for 1 h to oxidative stress induced by 100 μM H_2_O_2_ to evaluate protective properties against ROS or with 100 μM SIN-1 (peroxynitrite generator) to evaluate protective properties against NO in 5% CO_2_, 95% humidity, and 37 °C. Finally, the DCF-DA (for cells exposed to oxidative stress), Griess (for cells exposed to nitrosative stress), FHA (for cells exposed to oxidative stress) or MDA (for cells exposed to nitrosative stress) assays were performed.

1. Scavenging exogenous ROS or NO (RNS)

The ability to scavenge free radicals was tested by incubation in 100 μM H_2_O_2_ or only medium (depending on the test performed) or 100 μM SIN-1 for 1 h and then adding solutions of tested compounds (each at three concentrations: 10, 50 and 100 μM) in medium without serum and phenol red for 1 h. Finally, the DCF-DA (for cells exposed to oxidative stress), Griess (for cells exposed to nitrosative stress), FHA (for cells exposed to oxidative stress) or MDA (for cells exposed to nitrosative stress) assays were performed.

#### 2.1.5. DCF-DA Assay

DCF-DA (2′,7′-dichlorofluorescein diacetate) is a fluorescent dye that measures free radical levels. After diffusion into the cells, DCF-DA is deacetylated by esterases to a nonfluorescent compound that in the presence of ROS is oxidized to 2′,7′-dichlorofluorescein (DCF). The DCF-DA solution was prepared fresh before use by dissolving 1 mg of DCF-DA in 2.05 mL of 100% ethanol and diluting it in sterile filtered water, (BioReagent, suitable for cell culture, W3500, Sigma Aldrich, Saint Louis, MO, USA) to a final concentration of 10 μM. The intracellular level of free radicals was tested in two cases: after exposure to exogenous stress caused by reactive oxygen species (ROS) and without exposure to stress. Exogenous stress was induced with 100 μM H_2_O_2_ (hydrogen peroxide), an ROS generator. Solutions were made fresh before using the MEM without serum and phenol red. The ROS level was measured after further 1 h incubation with DCF-DA solution using a microplate reader (λex. = 485 nm, λem. = 535 nm).

#### 2.1.6. Griess Assay

The Griess assay was carried out to detect the presence of nitrite ions in the solution. Two reagents, 0.1% *N*-(1-naphthyl)ethylenediamine dihydrochloride and 1% sulfanilic acid, were combined in the same volume and mixed immediately before use. After cell regeneration and 24 h of incubation with tested compounds, the supernatant solution was removed. Cells were exposed to exogenous stress (100 μM SIN-1) or incubated only in the medium for 1 h. Then, the 150 μL of the solution was transferred to a new plate, and 20 μL of a mixture of Griess reagents and 130 μL of sterile-filtered water, (BioReagent, suitable for cell culture, W3500, Sigma Aldrich) was added for 30 min at room temperature (RT). Nitrite level was measured with the VarioScan Go at a wavelength of 548 nm.

#### 2.1.7. MDA Assay

Lipid peroxidation can occur as a result of oxidative damage by both ROS and RNS. Polyunsaturated lipids are susceptible to free radicals, leading to a specific reaction that produces end products such as malondialdehyde (MDA). After cell regeneration and 24 h of incubation with the compounds, the supernatant solution was removed. Cells were exposed to exogenous stress (100 μM SIN-1) or incubated only in the medium for 1 h. Cells were detached mechanically using a scraper, collected into tubes, added to 5 mL of 5% TCA, and prepared using an ultrasonic homogenizer. Then, the samples were centrifuged and the supernatant collected for further research. TBA was dissolved in 7.5 mL of glacial acetic acid to make 25 mL of TBA solution. MDA (0.1 M) was prepared by diluting 10 μL of 4.17 M MDA in 407 μL distilled water. Then, a 2 mM MDA standard was prepared, which was used to prepare a series of dilutions, which constituted the curve of standard MDAs. The tested supernatants and MDA dilutions were incubated in a water bath at 95 °C for 30 min with TBA solution. Then, to stop the reaction, the samples were transferred to ice for 10 min and centrifuged at 4000 rpm for 5 min. The supernatant was transferred to a 96-well plate, and absorbance at 530 nm was measured using the VarioScan Go microplate reader.

#### 2.1.8. FHA Assay

The fast halo assay (FHA) assessed DNA damage through a number of double-strand breaks (DSBs). After cell regeneration and 24 h of incubation with the compounds, the supernatant solution was removed. Cells were exposed to exogenous stress (100 μM H_2_O_2_) or incubated only in the medium for 1 h. Cells were detached mechanically using a scraper and collected into tubes. The plates were then washed with Hanks’ balanced salt solution (HBSS) to collect the remaining cells. The tubes were centrifuged (1000× *g*, 5 min), rinsed twice in PBS, and centrifuged again. The cell pellets were then placed in a 37 °C water bath and mixed with 130 μL of low-melting-point 1.25% agarose. This mixture of cells was placed on slides precoated with high-melting-point agarose, and then slides were covered with coverslips and put on a cooling block for approximately 10 min. After gelling the slides, the coverslips were removed and placed in a lysis buffer at 4 °C overnight. The next day, the preparations were transferred to an alkaline buffer (pH = 13) for 30 min and washed twice with a neutralizing buffer for 5 min. Finally, slides were stained with 5 μL of 4′,6-diamidino-2-phenylindole (DAPI) dye for 20 min in the dark and photographed using a fluorescence microscope. The analysis was performed for 3 independent replicates. Cells were harvested from the wells, and one slide was prepared for each concentration of each test compound. The entire preparation was analyzed by capturing images of cell nuclei. For each replicate, 10 randomly selected nuclei were evaluated.

#### 2.1.9. Statistical Analysis

In the performed bioassays, the normality of distribution was checked by the Shapiro–Wilk test, and the Levene test was used to check for equal variance. One-way ANOVA with Tukey’s post hoc test was also used. All analyses were performed using Statistica 13.1 software. A significance level of *p* < 0.05 was assumed in all tests. Based on the tests performed, the test power was calculated to be greater than 80%.

### 2.2. Interactions with Artificial Models of Cell Membranes

#### 2.2.1. Chemicals

Tris–EDTA buffer solution (pH 7.4) and 1,2-dipalmitoyl-*n*-glycero-3-phosphatidylcholine (DPPC) were purchased from Sigma-Aldrich. None of the compounds studied was soluble in water, so their chloroform (P.P.H. STANLAB, analytical grade) solutions were used for calorimetric experiments.

#### 2.2.2. Differential Scanning Calorimetry (DSC)

Calorimetric measurements were performed using a differential scanning calorimeter DSC 214 Polyma (Netzsch GmbH & Co., Selb, Germany) equipped with an Intracooler IC70 (Netzsch GmbH & Co., Selb, Germany) in the Laboratory of Elemental Analysis and Structural Research (Faculty of Pharmacy, Wroclaw Medical University). For each sample, 2 mg of phospholipid (DPPC) was dissolved in the appropriate amount of chloroform stock solution (5 mM) of the compounds studied (the compound:DPPC molar ratios in the samples were 0.06, 0.08, 0.10, and 0.12). The solvent was then evaporated under a stream of nitrogen gas. After that the residual solvent was evacuated under vacuum (Rotary evaporator, Büchy Poland, Warsaw, Poland) for 2 h. In this process, the phospholipid was transferred onto the dry film on the inner surface of the Eppendorf tube. Samples were hydrated by 20 μL of tris–EDTA buffer (pH 7.4). Hydrated mixtures of DPPC, compounds studied and buffer closed in Eppendorf tubes were heated (Labnet Dry Bath, Labnet International Inc., Edison, NJ, USA) to 10 °C higher than the main phase transition temperature of the phospholipid used (DPPC) and vortexed (neoVortex, neoLab, Heidelberg, Germany) until homogeneous dispersion was obtained. Then, the samples were transferred into aluminum sample pans (Concavus^®^, Netzsch GmbH & Co., Selb, Germany) and sealed. A pan of the same type filled with 20 μL of tris–EDTA buffer (pH 7.4) was employed as a reference. Measurements of the DPPC main phase transition were performed using the heat-flow measurement method at a heating rate of 1 °C per minute over a temperature range of 30–50 °C in a nitrogen dynamic atmosphere (25 mL/min). Data were analyzed offline using Netzsch Proteus^®^ 7.1.0 (Netzsch GmbH & Co., Selb, Germany) analysis software. The transition enthalpies were stated in (J/g). The measured heat was normalized per gram of lipid. The apparatus was calibrated using standard samples from calibration set 6.239.2–91.3 supplied by Netzsch (Netzsch GmbH & Co., Selb, Germany). All samples were weighed on a Sartorius CPA225D-0CE analytical balance (Sartorius AG, Göttingen, Germany) with a resolution of 0.01 mg.

### 2.3. Molecular Docking Studies

The crystal structures of human 15-lipoxygenase (4NRE), human serum albumin (2BXG and 2BXC) and α1-acid glycoprotein (3KQ0) were obtained from Protein Data Bank (http://www.rcsb.org, accessed on 20 June 2020). The structures of the studied compounds were optimized using DFT functional with B3LYP/6-311 + G (d.p) basic set [38,39,40]. Calculations were carried out using the Gaussian 2016 C.01 software package [41]. The molecular docking study was conducted using AutoDockVina 1.1.2 [42]. All the ligands and water molecules (except the iron ion with the coordinated two molecules of water in the 15-LOX structure) were removed, and then polar hydrogen atoms and Kollman charges were added to the protein structure using AutoDock Tools 1.5.6 [43]. To prepare the ligand molecules, partial charges were calculated, nonpolar hydrogens were merged, and rotatable bonds were assigned. Exhaustiveness values were set at 8, 16, 24, and 60. The center of the grid box was set according to the binding pocket site in the crystal structure. After the molecular docking. the ligand–receptor complexes were further analyzed using Discovery Studio Visualizer v.20 (https://www.3ds.com, accessed on 30 June 2020).

### 2.4. Spectroscopic Studies

#### 2.4.1. Fluorescence Quenching

Fluorescence spectroscopy analysis was performed using a Cary Eclipse 500 spectrophotometer (Agilent, Santa Clara, CA, USA). The concentrations of HSA and AAG were 1.0 × 10^−6^ mol/dm^3^. A 3 cm^3^ of a solution of each protein was titrated by successive additions of 1.0 × 10^−3^ mol/dm^3^ solution of the studied compounds. The molar ratio of compound to protein was 0–2 with 0.4 steps. Experiments were carried out at three temperatures: 297, 303, and 308 K in pH = 7.4 in phosphate buffer as a solvent. The quenching spectra were recorded at excitation and an emission wavelengths of 280 nm and 300–500 nm, respectively, with 5 mm path length. Binding site identification studies for HSA were indicated in the presence of the two site markers, phenylbutazone (PHB) and ibuprofen (IBP), as site I and II markers, respectively. Concentrations of HSA and site markers were 1.0 × 10^−6^ and 3.0 × 10^−6^ mol/dm^3^, respectively. Binding site studies for AAG were performed in the presence of quinaldine red (QR). The molar ratio of the compound to AAG/QR system was 0, 0.5, 1, 2, 3, 4, 5, 7, 9, 11, 13, 15. The quenching spectra were recorded at excitation and emission wavelengths of 500 nm and 510–700 nm, respectively, with a 5 mm path length at 297 K.

#### 2.4.2. Circular Dichroism Spectroscopy

Circular dichroism (CD) spectra were measured on a Jasco J-1500 magnetic circular dichroism spectrometer (Jasco International Co., Tokyo, Japan). Human serum albumin (HSA) and α-1-acid glycoprotein (AAG) powders were purchased from Sigma Aldrich. All measurements for the protein solutions in the absence and presence of the analyzed pyrrolo[3,4-*c*]pyrrole analogues **7a**–**7n** were made under simulated physiological conditions in pH 7.4 in phosphate buffer (in tablets, Sigma Aldrich) as a solvent at room temperature. The spectra were measured in the range of 205–250 nm at a scan rate of 50 nm/min, with a response time of 1 s and 10 mm path length. All of them were baseline-corrected (phosphate buffer was used as a baseline). The concentration of proteins was 1 × 10^−6^ mol/dm^3^, while for the analyzed compounds **7a**–**7n**, the concentration was equal to 1 × 10^−3^ mol/dm^3^. Experiments were performed with protein-to-ligand molar ratios of 1:0, 1:0.5, 1:1, 1:1, 1:3, and 1:5. A solution of each protein (3 cm^3^) was titrated by successive additions of analyzed compounds. The analysis of obtained results was made by CD Multivariate Calibration Creation and CD Multivariate SSE programs (Jasco International Co., Tokyo, Japan), with the conversion of protein concentrations for mean residue molar concentrations.

#### 2.4.3. FT-IR Measurements

The HSA and AAG proteins (Sigma Aldrich) were dissolved in an aqueous solution containing phosphate buffer (pH = 7.5) (Sigma Aldrich) to obtain 6×10^−4^ mol·dm^−3^ concentration. The compound solutions were prepared in methanol (Chempur, Karlsruhe, Germany) to achieve a 0.01 mol·dm^−3^ concentration. The protein solution was mixed with the studied compound at room temperature so that the sample has a molar ratio of protein to ligand equal to 1:1. Infrared spectra were recorded on a Nicolet iS50 FTIR (Thermo Scientific, Waltham, MA, USA) equipped with a deuterated triglycine sulfate (DTGS) detector and KBr beam splitter. The spectra were obtained using the attenuated total reflectance (ATR) method. Spectral data were recorded within the range of 3000 to 600 cm^−1^ with a resolution of 4 cm^−1^ and 100 scans per spectrum.

Analyses proceeded using Omnic 9.3.30 (Thermo Fisher Scientific Inc., Waltham, MA, USA) and OriginePro (OrigineLab Corporation, Northampton, MA, USA) software. The analysis of FTIR spectra was evaluated by Byler and Susi procedure [44]. After subtraction of the buffer background spectrum, the amide I peak was extracted and the second derivate made. This allowed for the selection of ingredient signals exactly related to the secondary structure. The peaks from the spectral range were responsible for shapes of α-helix (1650–1665 cm^−1^), β-sheet (1610–1640 cm^−1^), α-turn (1666–1673 cm^−1^), β-antiparallel (1675–1695 cm^−1^), and random coil (1640–1650 cm^−1^) were evaluated [44,45,46,47]. The fitting was conducted by Gaussian function. The self-deconvolution and curve fitting allowed us to determine the intensity and total area under peaks.

### 2.5. Computational Investigations

The Mannich base derivatives **7a**–**7c** and **7l**–**7m** were predicted for their possible pharmacokinetic (ADME), physicochemical, and drug-likeness properties using the SWISSADME server (http://www.swissadme.ch, date of access: 20 February 2023).

### 2.6. Chemistry

The synthesis and all experimental data describing the structure and physicochemical features of compounds **7a**–**7c** and **7l**–**7m** and all intermediates have been reported already [25].

## 3. Results

### 3.1. 15-Lipoxygenase (15-LOX) Inhibition Studies

#### 3.1.1. In Vitro 15-LOX Inhibition Assay

The impact of the six tested compounds (**7a**–**7c** and **7l**–**7n**) and zileuton (standard lipoxygenase inhibitor) on the activity of 15-LOX was evaluated with an incubation time of 5 min according to the procedure given by the kit manufacturer (760700). Afterwards, the IC_50_ values were calculated—concentrations at which 50% inhibition of enzyme activity occurred. All IC_50_ values are in Table 1. According to the obtained results, compounds **7a**–**7c**, **7l**, and **7m** appear to be better 15-LOX inhibitors than zileuton. The IC_50_ value calculated for **7n** was slightly higher than that of the reference drug. All investigated molecules revealed more or less the same 15-LOX inhibition activity.

#### 3.1.2. Molecular Docking Study

To determine how the compounds interact with 15-LOX, molecular docking studies were performed. The crystal structure of human 15-lipoxygenase with a substrate mimic inhibitor, PDB entry 4NRE [48], was used for calculations. The co-crystallized water and ligand molecules were removed, but the iron ion with the coordinated two molecules of water was retained. All compounds, **7a**–**7c**, **7l** and **7m**, were docked in the active site of 15-LOX. For all, a stable complex with 15-LOX was formed with binding affinity in the range of −7.3 kcal/mol to −9.7 kcal/mol for the best poses (Table 2). The best results were obtained for **7a** (among derivatives bearing phenylpiperazine moiety) and **7n** (among compounds with methylsulfonyl substituent).

**Figure 2:**
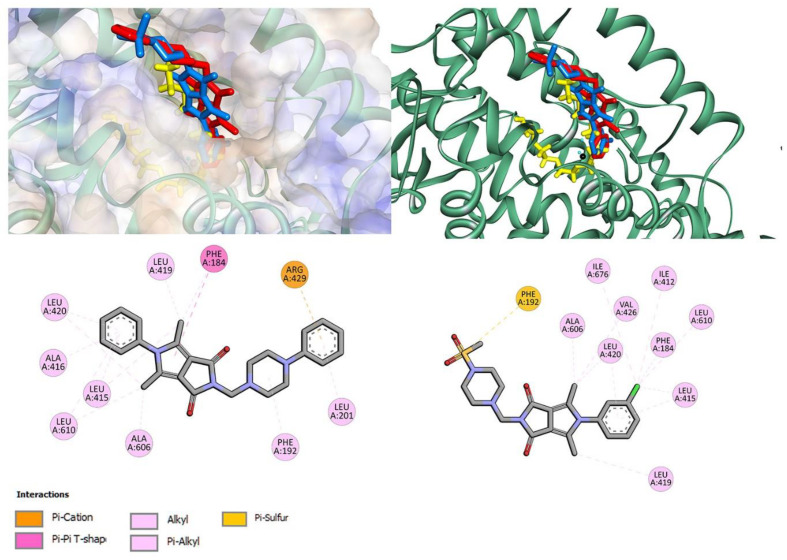
The docked pose of **7a** (red), **7n** (blue) into the active pocket site of 15-lipoxygenase (15-LOX) with substrate mimic inhibitor (yellow) and 2D interaction plot (**7a**—**left**, **7n**—**right**).

Figure 2 shows the interactions and position of compounds **7a** and **7n** in the active binding pocket. Both bind in one part of the U-shape channel. However, these compounds are shorter than native ligand (yellow). Compound **7a** does not directly interact with catalytic iron or amino acid residues coordinated to Fe^2+^: His373, His378, His553, Ile676. No hydrogen bonds are found. Several hydrophobic interactions are observed: π–alkyl with phenyl ring, π–alkyl and π–π with pyrrolo[3,4-*c*]pyrrole moiety, and π–alkyl and π–cation with the phenylpiperazine group. Details are presented in Figure 2. The phenyl ring of the pyrrolo[3,4-*c*]pyrrole moiety of compound **7n** is located near the metal region. Chloro substituent is involved in π interaction with Ile676 residue. The phenyl-pyrrolo[3,4-*c*]pyrrole moiety is well stabilized by hydrophobic contacts with residues Ala606, Leu420, Val426, Ile412, Phe184, Leu610, Leu415, and Leu419. At the other end of the molecule, methylsulfonylpiperazine moiety interacts with Phe192 via π–sulfur contact. The results from molecular docking studies suggest that the potential inhibitory activity is a non-redox mechanism and competes with a substrate to bind the active site [28,49,50,51,52].

### 3.2. Interactions with Artificial Models of Cell Membranes

**Figure 3:**
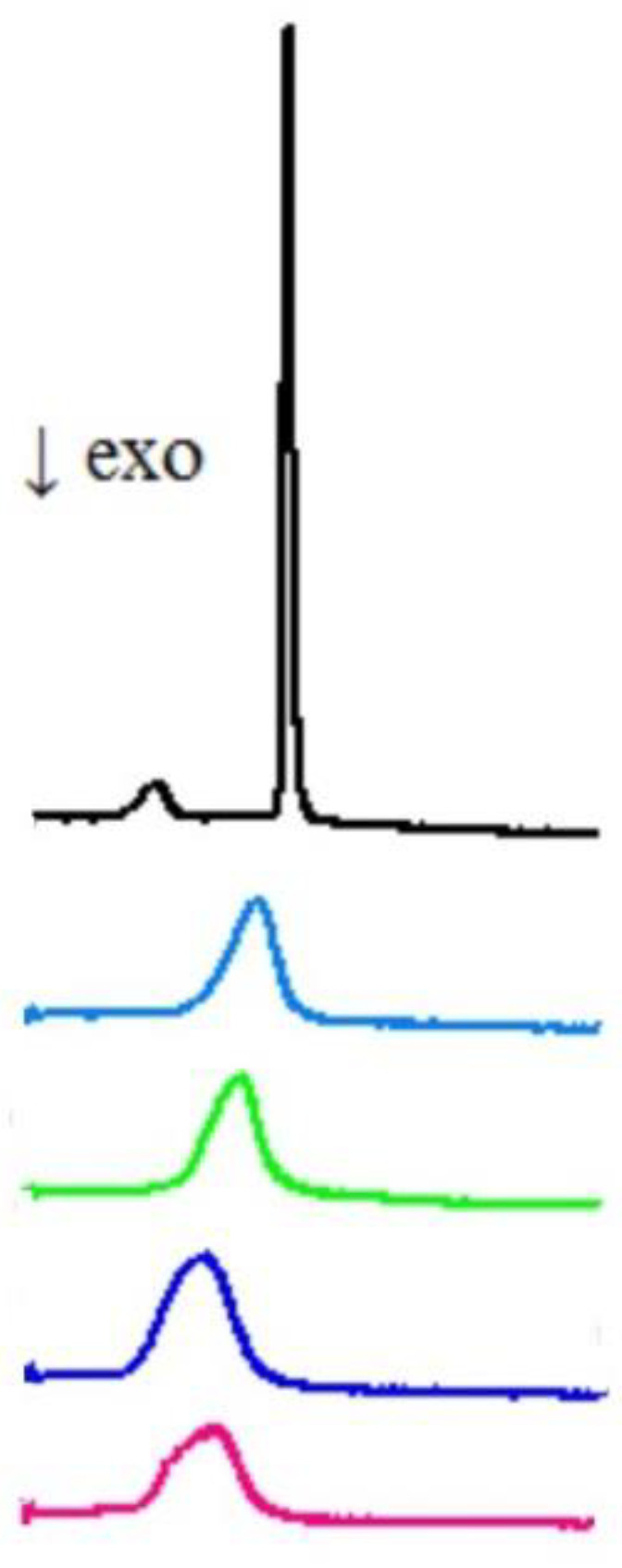
Thermograms (heat versus temperature) obtained for 1,2-dipalmitoyl-s*n*-glycero-3-phosphatidylcholine (DPPC) mixed with compound **7c** as well as for pure lipid (the first curve from the top—black). Curves in the figure represent the thermograms obtained for different molar ratios (studied compound DPPC—from the bottom: 0.12, 0.10, 0.08, 0.06, pure lipid). The exothermic direction in this graph is downward.

**Figure 4:**
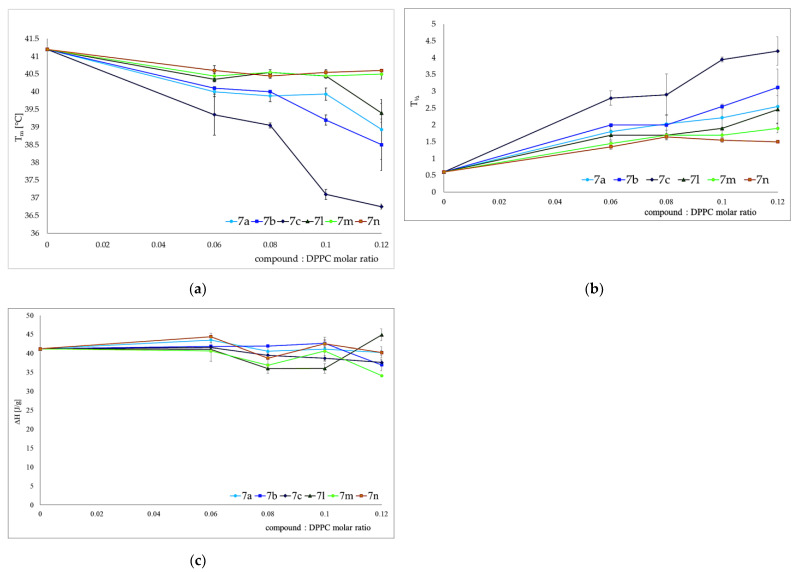
Influence of compounds on the parameters of 1,2-dipalmitoyl-s*n*-glycero-3-phosphatidylcholine (DPPC) main phase transition: temperature (**a**), peak width at half height (**b**) and enthalpy change (**c**). Bars represent standard deviations of four measurements. Where no error bars are shown, these were smaller than the symbols representing results.

Studying the interaction of potential drugs with biological membranes is quite important due to the fact that this interaction is very often a preliminary stage before absorption in the human body. Moreover, drugs may act on the surface of the cell membrane or have intracellular targets of action. However, these processes may be very complicated, and that is why, to study these mechanisms, simplified models of membranes are used. To investigate the interaction of the studied compounds with model membranes, we used multiple bilayers made of 1,2-dipalmitoyl-s*n*-glycero-3-phosphatidylcholine (DPPC) in a buffer solution (pH 7.4) as a model of the phospholipid membrane. Differential scanning calorimetry (DSC) was used as measurement method. The impact of the compound **7c** on the lipid thermal behavior is presented in Figure 3, showing the example thermograms of DPPC mixed with **7c** in different molar ratios. This compound decreased the main phase transition temperature (T_m_), and broadened the transition peaks (∆T_½_) the most among all tested compounds. The addition of all compounds caused the disappearance of the DPPC pretransition and concentration-dependent shift of the main transition temperature towards lower values, accompanied by a decrease in the transition peak area and the broadening of the peaks (Figure 4).

The interactions of studied compounds with artificial membrane models were also examined using different phospholipids, e.g., with shorter acyl chain: 1,2-dimyristoyl-sn-glycero-3-phosphocholine (DMPC). The dependence of the main DMPC phase transition temperature (Tm) and the peak transition half height (∆T½) on DMPC mixed with studied compounds for 0.1 compound:phospholipid molar ratio are shown in Table 3.

**Figure 5:**
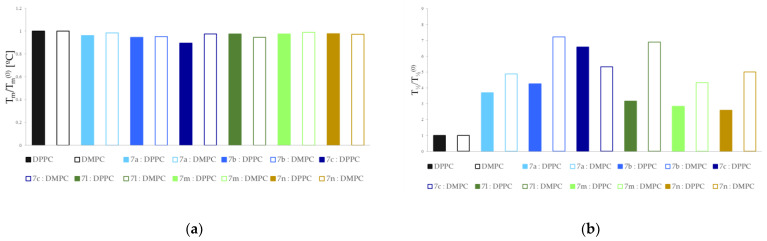
Relative main transition temperature (**a**) and transition peak width at half height (**b**) for 1,2-dipalmitoyl-s*n*-glycero-3-phosphatidylcholine (DPPC) and 1,2-dimyristoyl-sn-glycero-3-phosphocholine (DMPC) incorporated by compounds studied in 0.1 compound:phospholipid molar ratio.

The extent of compound-induced changes was larger in DMPC than in DPPC (Figure 5). This may be due to the fact that DMPC is a phospholipid with shorter acyl chains.

All derivatives added to model membranes influenced the thermotropic properties of phospholipids in a concentration-dependent manner. All examined compounds decreased the main transition temperature (Tm), increased transition peak width at half height (T½) by broadening the transition peaks, and decreased the enthalpy (ΔH) of the main phase transition. The character of the changes may allow us to conclude that interactions between phospholipid molecules in the gel state became weaker in the presence of the compounds, and that lipid polar heads as well as hydrocarbon chain regions were affected by the compounds (according to the standard interpretation of calorimetric data proposed by Jain and Wu in 1977) [53]. In o the case of the parameter changes of DPPC gel–liquid crystalline phase transition studied here, the most pronounced effects were found in the presence of compounds **7c**, **7b** and **7a**.

### 3.3. Antioxidant Activity within Cells

Oxidation mediators such as lipid peroxides and nitric oxide affect cell death. The key enzyme that catalyzes lipid peroxide formation is 15-lipoxygenase-1 (15-LOX-1). Inhibition of 15-LOX-1 may interfere with regulated cell death in inflammatory processes by inhibiting NO formation and lipid peroxidation, which may be related to effects on nuclear factor κB signaling [16].

At the same time, the increased level of reactive oxygen and nitrogen species may induce growth in lipoxygenase activity. Metabolic changes lead to ROS and RNS formation. Excess ROS causes oxidative stress, and RNS causes nitrosative stress. We induced oxidative stress with 100 μM H_2_O_2_ and nitrosative stress with 100 μM SIN-1.

The results of the DCF-DA assay are shown in Table 4, where values less than 1 indicate a decrease in ROS level compared to the control, while values higher than 1 indicate a higher ROS level. Protective properties of compounds against oxidative stress were observed for all compounds—the level of oxygen free radicals was reduced compared to control (1 h incubation of NHDF cells with 100 μM H_2_O_2_; without tested compounds).

Analyzing the level of free radicals after treatment with compounds and without H_2_O_2_ (compared to the control—NHDF cell culture in a full DMEM culture), it should be noted that all tested compounds influenced the decrease in the level of free radicals after 24 h incubation. In the case of 1 h incubation of cells with compounds, the reduction in ROS formation was observed for **7a** over the whole range, in 100 μM for **7b** and **7c**, and 100 and 50 μM for **7l**.

Regardless of the incubation time (24 h or 1 h) of cell cultures with compounds **7a**–**7c**, greater scavenging of ROS was observed after using higher concentrations. However, in the case of compounds **7l**–**7n**, the reduction was the strongest after incubation with the lowest concentration tested.

Griess assay results are shown in Table 5, where values less than 1 indicate a decrease in nitrite ion level compared to the control. Higher values indicate that the nitrite ion level increased upon the addition of the compounds. For example, when NHDF cells were treated for 24 h with the compounds and next for 1 h with 100 μM SIN-1, a statistically significant reduction in RNS level (4.9–22.5%) was observed for all compounds at all concentrations compared to the control (NHDF cell culture with 100 μM SIN-1; without compounds).

After 24 h of incubation of NHDF cells with the tested compounds, it was observed that all compounds caused a reduction in the level of nitrite ions compared to the control (culture without tested compounds). During a 1 h incubation with the test compounds, NO reduction was observed for compounds **7c** and **7l**–**7n** over the entire concentration range (however, this reduction was not statistically significant compared to controls).

Regardless of the incubation time (24 h or 1 h) of cell cultures with compound **7a**, greater NO uptake was observed after using higher concentrations. In the case of the remaining compounds, the reduction was strongest after incubation with the lowest tested concentration.

Oxygen free radicals affect the damaged strand of DNA. Therefore, the effect of the tested compounds on the protective effect against DNA strand breaks was assessed using the fast halo assay (FHA).

The results of the FHA assay are shown in Table 6, where values less than 1 indicate a reduction in the number of DNA double breaks compared to the control, while values higher than 1 indicate a higher number of DNA breaks. Protective properties of the compounds against DNA strand damage due to oxidative stress were observed for all compounds—the incidence of DNA damage was reduced compared to the control (1 h incubation of NHDF cells with 100 μM H_2_O_2_; no tested compounds).

After 24 h of incubation of the NHDF cells with the test compounds, it was observed that all compounds caused a statistically significant reduction in the number of DNA breaks compared to the control (culture without test compounds). During a 1 h incubation with the test compounds, there was a reduction in DNA damage for **7a** at 100 and 10 µM, over the whole concentration range for **7b**, and at 100 µM for **7c**, while the only statistically significant reduction in DNA strand breaks was observed at 1 µM for **7l**.

At the same time, a correlation was found between the reduction in DNA strand breaks and the level of ROS. Regardless of the incubation time, for compounds **7a**–**7c**, a decrease in DNA breaks was observed with increasing concentration. However, in the case of compounds **7l**–**7n**, the lowest number of DNA breaks was observed at the lowest tested concentration.

Lipid peroxidation can contribute to many diseases deriving from inflammation. It is well known that the level of NO impacts the degree of lipid peroxidation. Therefore, it was checked whether the tested compounds affected the reduction of lipid peroxidation by performing the MDA assay.

The results of the MDA assay are shown in Table 7, where values less than 1 indicate a reduction in lipid peroxidation compared to the control. In contrast, values higher than 1 indicate higher lipid peroxidation. Protective properties of the compounds against lipid peroxidation due to the level of NO were observed for all compounds—lipid peroxidation was reduced compared to the control (1 h incubation of NHDF cells with 100 μM SIN-1; no tested compounds).

A statistically significant reduction in lipid peroxidation was observed across the concentration range after applying compounds **7a** and **7b**, regardless of the incubation time.

An association of a reduction in lipid peroxidation with a reduction in NO levels was observed. Despite the incubation time, a decrease in lipid peroxidation was observed for compound **7a** with increasing concentration. However, in the case of the remaining compounds tested, the lowest lipid peroxidation was observed at the lowest tested concentration.

### 3.4. Human Serum Albumin (HSA) and Alpha-1-Acid Glycoprotein (AAG) Ligand-Binding Assay

The interactions of drugs with plasma proteins have a splendid impact on their pharmacokinetic parameters in vivo. For this reason, we carried out experiments aimed at the evaluation of the binding mode of Mannich base derivatives of pyrrolo[3,4-*c*]pyrrole (**7a**–**7c**, **7l**–**7m**) with human serum albumin (HSA) and alpha-1-acid glycoprotein (AAG). HSA and AAG are the most abundant blood proteins and are significantly involved in the binding and distribution of xenobiotics. The molecular interactions between the compounds and target plasma proteins can be traced using such optical techniques as CD, FT-IR, UV-vis or fluorescence spectroscopy [34,35].

#### 3.4.1. Fluorescence Quenching, Binding Constants, Site Markers and Thermodynamic Studies

**Figure 6:**
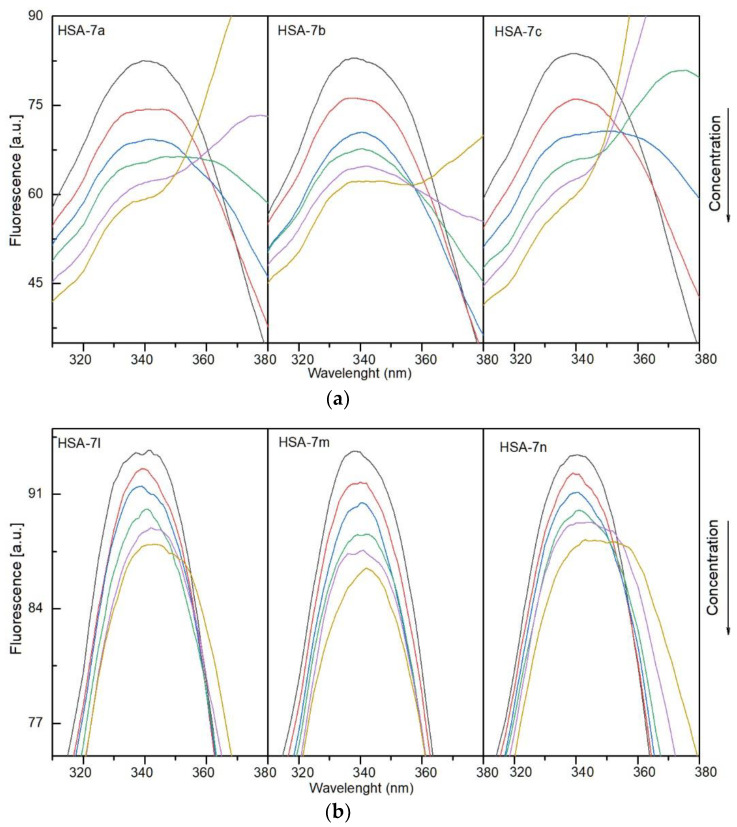
(**a**). Fluorescence quenching spectra of human serum albumin (HSA) in the presence of different concentrations of compounds **7a**–**7c** (T-297 K, λ_ex_ = 280 nm). The concentration of HSA was 1.0 µM, the concentration of **7a**–**7c** 0, 0.4, 0.8, 1.2, 1.6, and 2.0 µM. (**b**). Fluorescence quenching spectra of human serum albumin (HSA) in the presence of different concentrations of compounds **7l**–**7m**. (T-297 K, λ_ex_ = 280 nm). The concentration of HSA was 1.0 µM, the concentration of **7l**–**7m** 0, 0.4, 0.8, 1.2, 1.6, and 2.0 µM.

**Figure 7:**
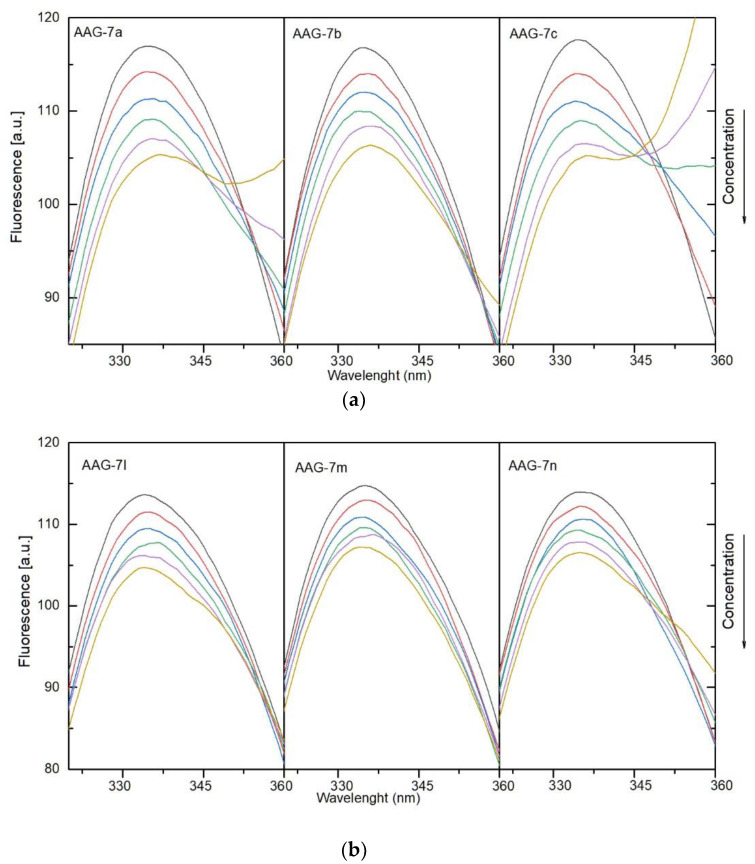
(**a**). Fluorescence quenching spectra of alpha-1-acid glycoprotein (AAG) in the presence of different concentrations of compounds **7a**–**7c** (T-297 K, λ_ex_ = 280 nm). The concentration of AAG was 1.0 µM, the concentration of **7a**–**7c** 0, 0.4, 0.8, 1.2, 1.6, and 2.0 µM. (**b**). Fluorescence quenching spectra of alpha-1-acid glycoprotein (AAG) in the presence of different concentrations of compounds **7l**–**7m** (T-297 K, λ_ex_ = 280 nm). The concentration of AAG was 1.0 µM, the concentration of **7l**–**7m** 0, 0.4, 0.8, 1.2, 1.6, and 2.0 µM.

To determine the nature of interactions of the compounds with human serum albumin and alpha-1-acid glycoprotein and complex formation, fluorescence spectroscopy was used. Solutions of **7a**–**7c** and **7l**–**7n** were added to a solution of plasma protein. Fluorescence quenching was observed. The results showed that the fluorescence intensity of HSA and AAG decreased with a successive increase in concentration of the studied compounds. This indicates interaction between protein and **7a**–**7c**, **7l**–**7m**. The quenching spectra are presented in Figure 6a,b for HSA and Figure 7a,b for AAG. A shift in the maximum of the emission peak was also observed for both systems with HSA and AAG. A more evident shift was detected for the interactions with albumin. This suggests that the microenvironment around the chromophore of proteins had changed [54].

Fluorescence quenching does not mean the formation of a complex with the protein. This can only be caused by collisions. To determine if the interactions are dynamic or static, leading to the formation of a stable complex, the measurements were carried out at three temperatures: 297, 303, and 308 K. The obtained results were analyzed using the classical Stern–Volmer Equation (1), after correction due to the inference filter effect (2):

(1) $$\frac{F_{0}}{F}=1+k_{q}\tau \left[Q\right]=1+K_{\mathrm{SV}}\;$$

(2) $$F_{\mathrm{corr}}=F_{\mathrm{obs}}10^{\frac{\left(A_{\mathrm{ex}}+A_{\mathrm{em}}\right)}{2}}$$

where F_0_ and F are the steady-state fluorescence intensities at the maximum wavelength in the absence and presence of a quencher, respectively, k_q_ the quenching rate constant of the biomolecule, τ_0_ the average lifetime of the biomolecule, [Q] the quencher concentration, K_sv_ the Stern–Volmer constant, F_corr_ and F_obs_ the corrected and observed fluorescence intensities, respectively, and A_ex_ and A_em_ the absorbance values at excitation and emission wavelengths, respectively.

A linear regression fit was used to compute the value of K_sv_. Then, assuming the average lifetime of the fluorophore in the excited state for a biomolecule as 10^−8^ s [55], the quenching rate constant (k_q_) was obtained. The calculated results are listed in Table 8 for HSA and Table 9 for AAG. For all systems, with HSA and AAG, (k_q_) had a value greater than 10^12^. It can also be observed that k_q_ (and K_SV_) values are higher for complexes with HSA, indicating that the compounds have a stronger affinity towards the excited fluorophores of HSA than AAG. However, for both, the quenching rate constant is much higher than 2 × 10^10^ dm^3^·mol^−1^·s^−1^ [56,57], i.e., higher than the maximum value in an aqueous solution for the dynamic quenching mechanism. This suggests a static mechanism, i.e., the formation of a complex with the protein. Measurements at different temperatures also indicate a static mechanism. As shown in Table 8 and Table 9, K_SV_ and k_q_ decrease with increasing temperature. This clearly indicates that compounds **7a**–**7c** and **7l**–**7m** quench the fluorescence of HSA and AAG through a static quenching mechanism rather than a dynamic one.

**Figure 8:**
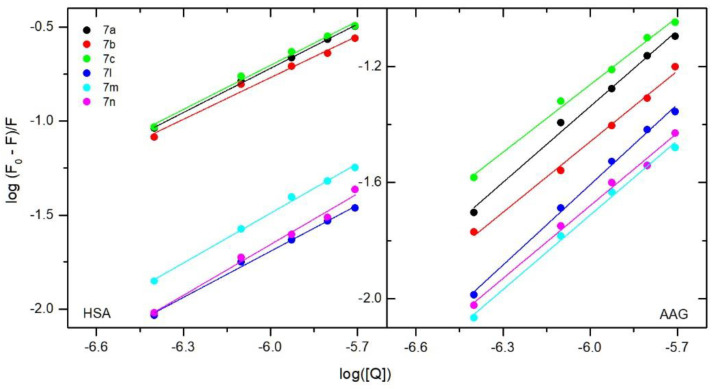
Double-logarithm-regression plots for quenching of human serum albumin (HSA) (**left**) and alpha-1-acid glycoprotein (AAG) (**right**) by compounds **7a**–**7c** and **7l**–**7m**.

To estimate the stability of complexes with HSA and AAG, the binding constants were calculated. A double-log-regression curve was used to fit the experimental data according to Equation (3):

(3) $$\log\frac{F_{0}-F}{F}={\mathrm{logK}}_{b}+\mathrm{nlog}\left[Q\right]$$

where F_0_ and F are the fluorescence intensities at the maximum wavelength (after correction due to the inference filter effect) in the absence and presence of a quencher, respectively, and [Q] is the quencher concentration. The plot is presented in Figure 8. A good linearity relationship can be observed between log [(F_0_ − F)/F] and log [Q]. The calculated values of binding constants K_b_ are collected in Table 8 and Table 9. Interactions with human serum albumin K_b_ were from 1.66 × 10^3^ for 7l to 9.77 × 10^3^ dm^3^·mol^−1^ for 7c. However, the differences between the tested compounds were not significant. The structural modification does not affect the binding constant much. K_b_ values of the compounds show that the interactions with HSA are moderate. Similar values were obtained for popular anti-inflammatory drugs [58] or compounds with biological activity [33,59,60,61,62,63,64]. The calculated K_b_ values for interactions with alpha-1-acid glycoprotein are close to interactions with HSA. This means similar ability to be released from the complex into the bloodstream.

Carrying out fluorescence quenching measurements at three temperatures allowed for the calculation of thermodynamic parameters: enthalpy change (ΔH°), entropic change (ΔS°), and free energy change (ΔG°). The following Equations (4) and (5) were used:

(4) $${\mathrm{logK}}_{b}=-\frac{\Delta H^{{}^{\circ}}}{\mathrm{RT}}+\frac{\Delta S^{{}^{\circ}}}{R}$$

(5) $$\Delta G^{{}^{\circ}}=\Delta H^{{}^{\circ}}-T\Delta S^{{}^{\circ}}=-{\mathrm{RTlnK}}_{b}$$

where K_b_ is the binding constant and R the universal gas constant. The results are listed in Table 8 and Table 9. For all interactions, the ΔG° values are negative. This indicates that the binding process is spontaneous. The calculated values for ΔH° and ΔS° are also negative, indicating that van der Waals forces and/or hydrogen bonds are the main interaction types in the binding process.

Additionally, to confirm the binding **7a**–**7c**, **7l**–**7m** to the active sites of HSA and AAG, fluorescence spectroscopy experiments were carried out in the presence of markers: phenylbutazone (PHB), ibuprofen (IBP) for HSA, and quinaldine red (QR) for AAG. For interactions with albumin, the obtained data were analyzed by Equation (3). Binding constants in the presence of **7a**–**7c**, **7l**–**7m**, and site markers were calculated and compared with sK_b_ without PHB and IBP. Results are presented in Table 10.

**Figure 9:**
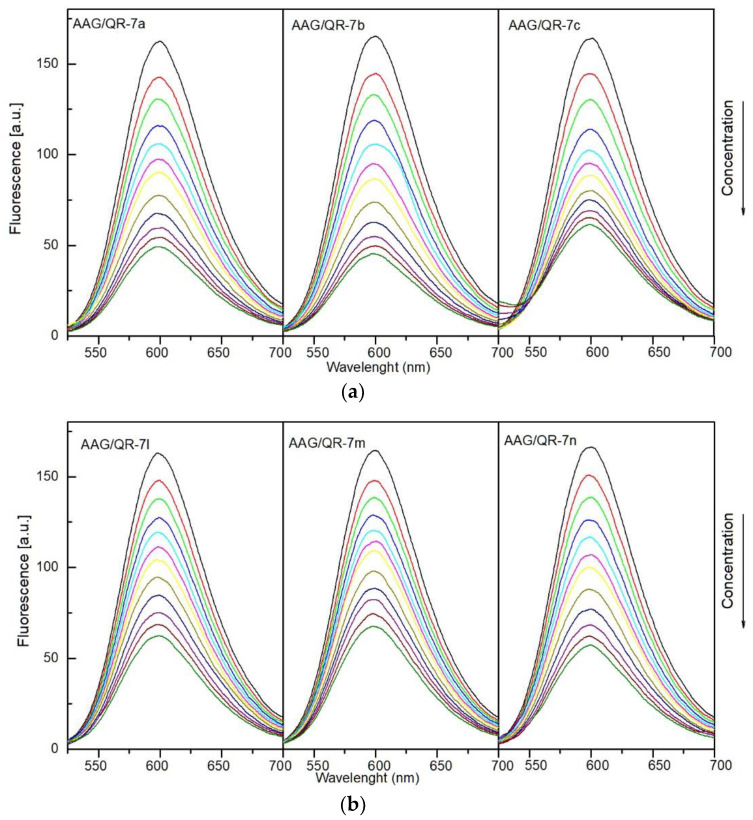
(**a**) Fluorescence quenching spectra of the alpha-1-acid glycoprotein (AAG)/QR system in the presence of different concentrations of compounds **7a**–**7c** (T-297 K, λex = 500 nm). The concentration of **7a**–**7c** were 0, 0.5, 1, 2, 3, 4, 5, 7, 9, 11, 13, 15 µM. (**b**) Fluorescence quenching spectra of alpha-1-acid glycoprotein (AAG)/QR system in the presence of different concentrations of compounds **7l**–**7m** (T-297 K, λex = 500 nm). The concentration of **7l**–**7m** is 0, 0.5, 1, 2, 3, 4, 5, 7, 9, 11, 13, 15 µM.

The K_b_ value of all tested compounds decreased in the presence of both PHE and IBP markers. This indicates that the studied molecules can interact with HSA in the pocket occupied by both phenylbutazone (site 1) and ibuprofen (site 2). For compounds, **7b** and **7m** with an n-butyl substituent, site 1 is slightly more preferred. For molecules with phenyl or m-Cl-phenyl substituent in position 5 of the pyrrolo[3,4-*c*]pyrrole moiety (**7a**, **7c**, **7l**, **7n**), site 2 is slightly more preferred. However, the differences in the K_b_ values are not very significant. Figure 9a,b present fluorescence quenching spectra of the AAG/QR system after addition of **7a**–**7c**, **7l**–**7m**. Quinaldine red shows strong fluorescence after binding to a protein, while the fluorescence in the unbound form is low [65,66]. After adding more portions of **7a**–**7c** and **7l**–**7m**, the fluorescence intensity decreases. This indicates displacement of QR from the AAG/QR complex and binding of test compounds to the site occupied by quinaldine red.

#### 3.4.2. Circular Dichroism Spectra

**Figure 10:**
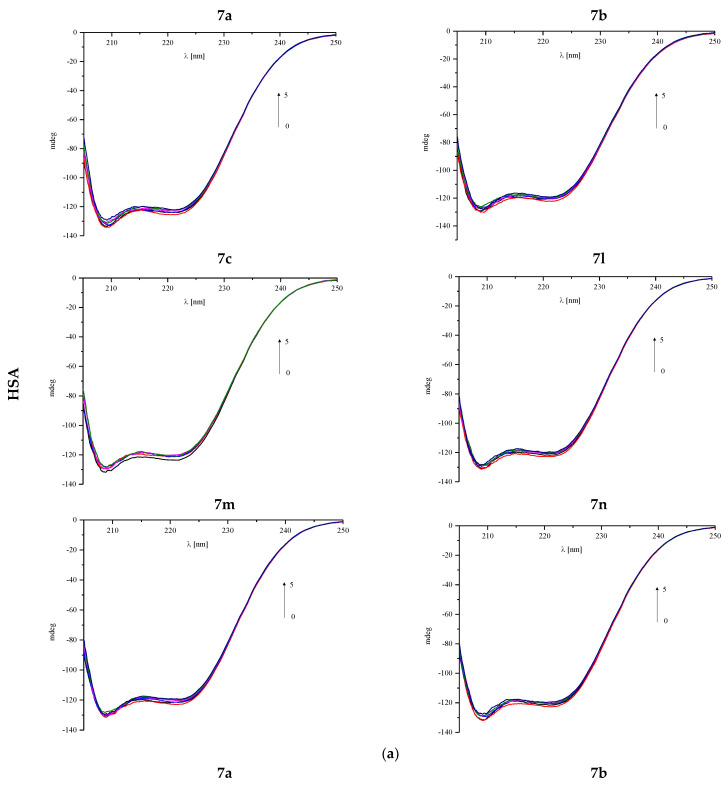

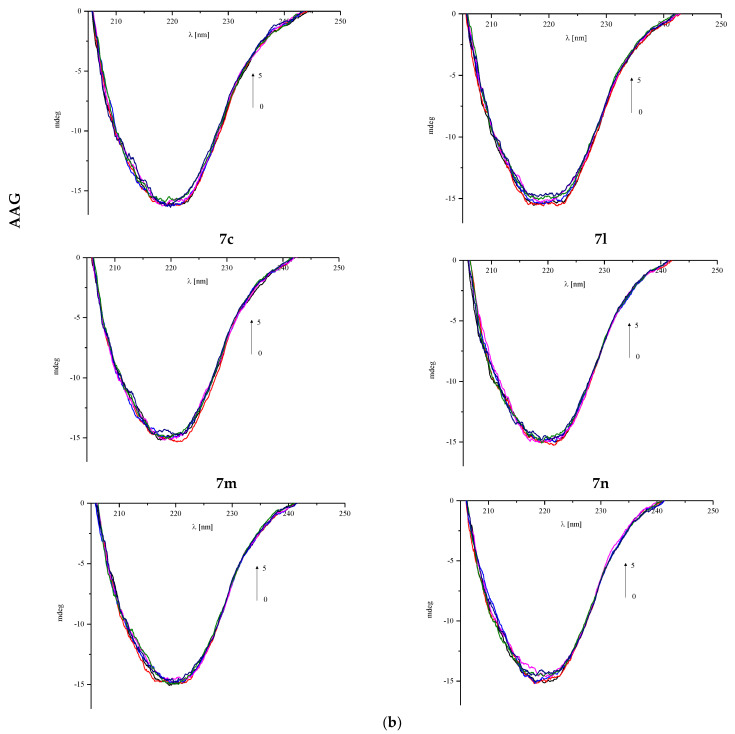
(**a**) Circular dichroism spectra of human serum albumin (HSA) in the absence and presence of analyzed compounds **7a**–**7c** and **7l**–**7n**. (**b**) Circular dichroism spectra of alpha-1-acid glycoprotein (AAG) in the absence and presence of analyzed compounds **7a**–**7c** and **7l**–**7n**.

The interaction between the protein and the tested compound can cause changes in the secondary structure, which can be observed in the circular dichroism (CD) spectra [34]. Characteristic bands are present for specific structures such as α-helix (two negative peaks near 209 and 220 nm) or β-sheet (the negative band around 215 nm) [32]. Therefore, the CD spectra for plasma proteins HSA and AAG in the absence and presence of compounds **7a**–**7c** and **7l**–**7n** were measured (Figure 10a,b). The changes after adding every portion of the pyrrolo[3,4-*c*]pyrrole analogues from 1:0 to 1:5 protein to analyzed compound molar ratios were observed. Obtained results were analyzed by the CD Multivariate SSE program, and are summarized in Table 11 and Table 12.

Obtained CD spectra are typical for analyzed proteins. For has, two negative α-helix bands are present (Figure 10a). The changes observed in the spectrum during the addition of successive portions of the test compounds are small. There is a slight reduction in the percentage of α-helix (Table 11). It can be assumed that the analyzed compounds interact with the albumin molecule; however, their presence does not destabilize the protein structure. For all analyzed compounds, the observed changes in α-helix content do not exceed 1.5% and are similar for all of them.

One negative band near 220 nm is observed in AAG spectra (Figure 10b). Increasing the concentration of the analyzed compounds slightly affects the course of the spectrum, which is also confirmed by the analysis of CD results by the CD Multivariate SSE program (Table 12). The structure of the protein consists of more than 30% of β-sheet and about 20% of α-helix. These are the two dominant structures in this protein. Changes in the percentage of both forms after adding a fivefold excess of test compounds do not exceed 1% (Table 12). The α-helix percentage decreases in favor of the β-sheet. To sum up, it can be concluded that the binding of pyrrolo[3,4-*c*]pyrrole analogues to the studied protein does not affect significantly its structure.

#### 3.4.3. Fourier-Transform Infrared Spectroscopic Measurements

**Figure 11:**
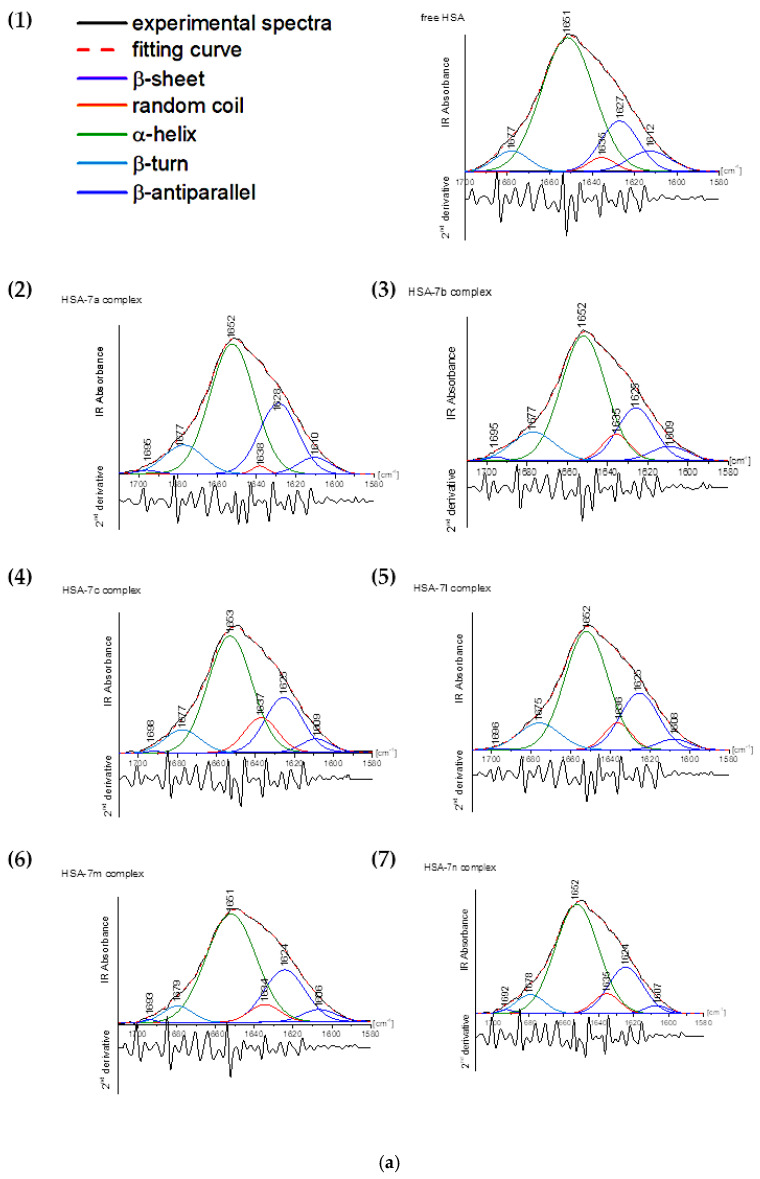

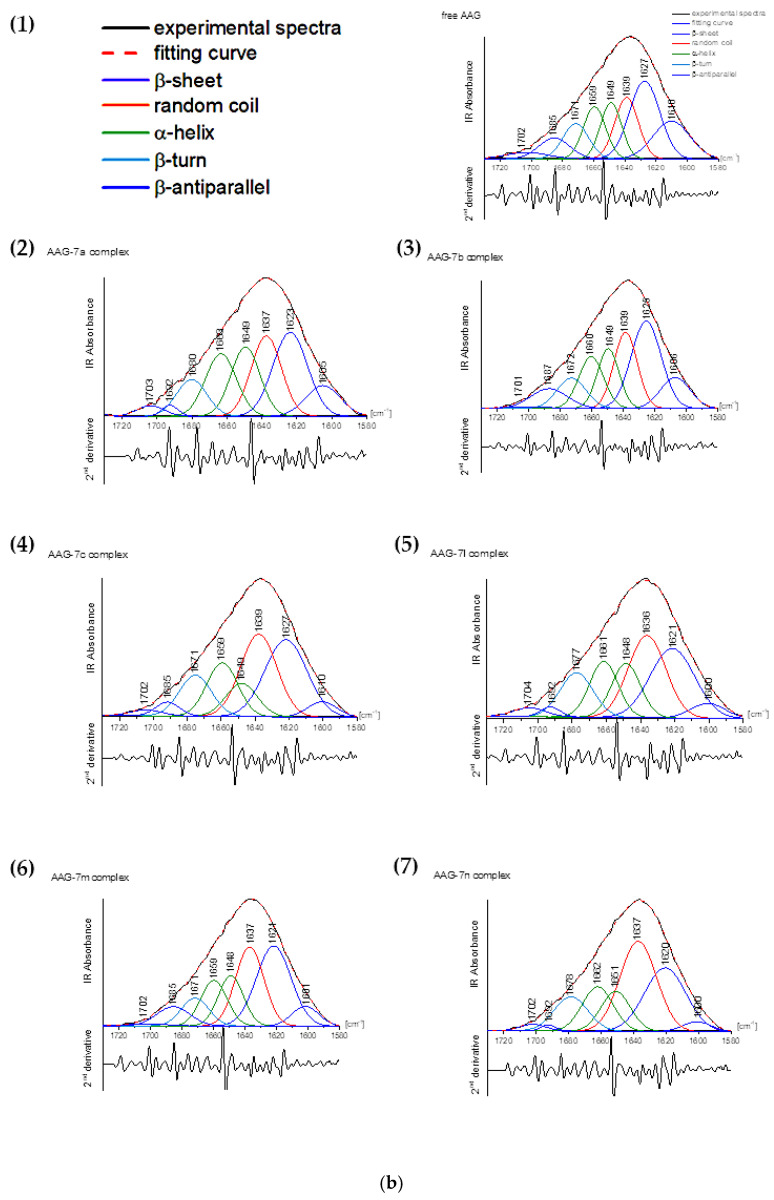
(**a**) Curve-fitted amide I region for: (**a1**) free human serum albumin (HSA) and HSA complex with (**a2**) **7a**, (**a3**) **7b**, (**a4**) **7c**, (**a5**) **7l**, (**a6**) **7m**, and (**a7**) **7n** compounds. (**b**) Curve-fitted amide I region for: (**b1**) free alpha-1-acid glycoprotein (AAG) and AAG complex with: (**b2**) **7a**, (**b3**) **7b**, (**b4**) **7c**, (**b5**) **7l**, (**b6**) **7m**, and (**b7**) **7n** compounds.

Fourier-transform infrared spectroscopy (FT-IR) is a fast and nondestructive method widely used in medicine and pharmacy fields. So far, it has been applied to evaluate biological fluids such as tissues, cells, lipids, and DNA, and even whole organisms such as fungi, bacteria, and viruses [67,68,69,70,71,72,73,74]. This method is also efficacious to control the secondary structure of peptides and proteins to examine drug binding to protein plasma [75,76,77,78]. The most abundant information range of wavenumber is 1700 to 800 cm^−1^. Usually, amide I and amide II peaks are observed, but amide III to VII may also be seen. Amide I is the most common signal to study the conformation changes and accrues by the C=O stretching vibration with the contribution of C–N stretching, C–C–N deformation, and N–H bending in-plane [44,67,68,69,70,71,72,73,74,79]. Nonetheless, the amide I signal is more sensitive than other bonds to changes in secondary structure; therefore, it is more often chosen for this type of research [47]. The energy of hydrogen bonds in proteins and complexes with pharmacologically active molecules affects the frequency of absorption of C=O vibrations, which corresponds to a change in the secondary structure of proteins. It has been shown that hydrogen bonding reduces the frequency of stretching vibrations and increases the frequency of bending vibrations [79]. The position of amide I for free HSA was detected at 1651 cm^−1^, and for free AAG was at 1635 cm^−1^. These values are similar to those earlier described in the literature [80,81,82,83]. Rather a major difference in the position of the amide I band results from a significant dissimilarity in the secondary structure of these proteins. The self-deconvolution of amide I bonds for free plasma proteins and their complexes with investigated pyrrolo[3,4-*c*]pyrrole derivatives are presented in Figure 11a,b.

The conformation of HSA is mostly dominated by αhelix (64.55%), approximately 25.42% of β-sheet, and less than 1% of β-antiparallel shape (Table 13, Figure 11a).

In contrast, AAG is structurally a more diverse protein. The β-sheet structure predominates, which is about 40% in the protein solution. The α-helix is 25.02% and the random coil structure is about 15%. The structure of β-turn and β-antiparallel makes up about 10% (Table 14).

A slight shift in the maximum of the amide I band after the addition of the studied compounds indicates their binding to HSA. Moreover, the band’s shape variation was observed, justifying the perturbation in the secondary structure. Generally, the interaction of pyrrolo[3,4-*c*]pyrrole-based Mannich bases with HSA caused a reduction in α-helix for **7m**, and **7n**, and for **7a**–**7c**, this was only about 5% to 8%, but for **7l** it was more than 10% (Table 13, Figure 11a). A minor increase in the participation of the β-sheet structure was observed, to about 1% for **7b**, **7c**, **7l** and **7n** compounds and to 5% for the **7a** analogue. The rise of β-turn was detected for all pyrrolo[3,4-*c*]pyrrole derivatives except for **7m**, where 1% less β-turn was noticed. The random coil fraction grew to 3–4% for **7b**, **7c** and **7l**–**7n** and fell 1% after interaction with **7a**. The binding of the derivatives to HSA did not impact the β-antiparallel structure.

The binding pyrrolo[3,4-*c*]pyrrole analogues with AAG forced decomposition of β-sheet. The compounds **7l** and **7n** had the most significant influence to reduce about 10% of β-sheet (Table 14, Figure 11b). The share of β-antiparallel was smaller than native protein, for **7n** it was approx. 7%, for **7a**, **7c**, and **7l** about 6%, and for **7b** and **7m** about 1%. A similar tendency was observed for increasing β-turn structure after the addition of **7a**, **7c**, **7l**, and **7n** to the protein solution. Only AAG complexes with **7b** and **7m** had little reduction (0.5–1.5%) of β-turn percentages. The α-helix structure did not change too much. Surprisingly, an increase of up to 2% was detected for complexes **7b**, **7c**, and **7l**–**7n**, and an increase of up to 5% was observed for the **7a** complex. The unique structural changes in AAG were previously described by Nishi et al., who reported that AAG changes the β-form to α-helix after micelle and liposome interaction [84,85]. The largest increase was observed in the random coil structure for **7n**: 16% compared to native AAG. For **7c** and **7l**, the changes were around 10%, and below for the remaining analogues.

The interaction of investigated pyrrolo[3,4-*c*]pyrrole derivatives with HSA plasma protein induced destabilization of the protein α-helix in favor of a random coil structure. The exception was compound **7a**, the presence of which caused the growth of β-forms. In the case of complexes with AAG, the magnification of random coil structure occurred with a decrease in the β-sheet and β-antiparallel shape.

#### 3.4.4. Site Markers and Molecular Docking Studies

To identify how studied compounds interact with two plasma proteins, HSA and AAG, molecular docking analyses were done. The crystal structures of human serum albumin with co-crystalized ibuprofen (IBU) and phenylbutazone (PHB) (PDB entries 2BXG and 2BXC [86]) and human alpha1-acid glycoprotein (PDB entry 3KQ0 [87]) were used for calculations. The co-crystallized water and ligand molecules were removed. For HSA interaction studies, the docking area was set to the two positions of ibuprofen (IIIA, IIA–IIB) and phenylbutazone (IIA) [86]. For all compounds, a stable complex with HSA was formed, with negative binding affinity in the range from −7.6 kcal/mol to −8.9 kcal/mol for the best poses (Table 15).

**Figure 12:**
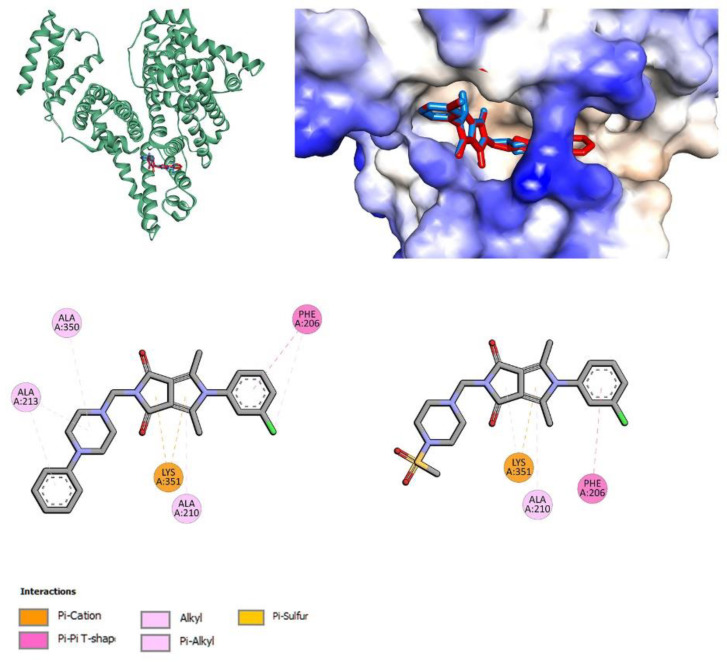
The docked pose of **7c** (red) and **7n** (blue) into the pocket of human serum albumin (HSA) (IIA–IIB), and 2D interaction plot (**7c**—**left**, **7n**—**right**).

The studied compounds can bind to both IBU and PHB binding sites. Of the two places occupied by ibuprofen, the location in the middle of albumin (IIA–IIB) is preferable. Molecular docking results also showed that binding affinity for molecules with phenyl or m-Cl-phenyl substituent in position 5 of the pyrrolo[3,4-*c*]pyrrole core (**7a**, **7c**, **7l**, **7n**) is more negative for interactions into the pocket of ibuprofen location (IIA–IIB). For compounds with *n*-butyl substituent (**7b**, **7m**), the pocket of phenylbutazone (IIA) is preferable. The obtained results correlate well with our fluorescence spectroscopy studies. Molecules **7c** (series **a**–**c**) and **7n** (series **l**–**n**) show the strongest interactions with HSA (Table 15). Figure 12 presents the interactions and position of compounds **7c** and **7n** into active binding pocket IIA–IIB.

No hydrogen bonds were found. The 3-chlrophenyl substituent in position 5 of the pyrrolo[3,4-*c*]pyrrole moiety interacts with Phe206 residue via π–π contact. Lys351 and Ala210 residues are involved in π–cation and π–alkyl interactions with the pyrrolo[3,4-*c*]pyrrole group. Compound **7c** is additionally stabilized by interactions with the phenylpiperazine moiety. The compounds also formed a stable complex with alpha-1-acid glycoprotein. Binding affinity was −8.1 kcal/mol to −9.7 kcal/mol for the best poses (Table 15).

**Figure 13:**
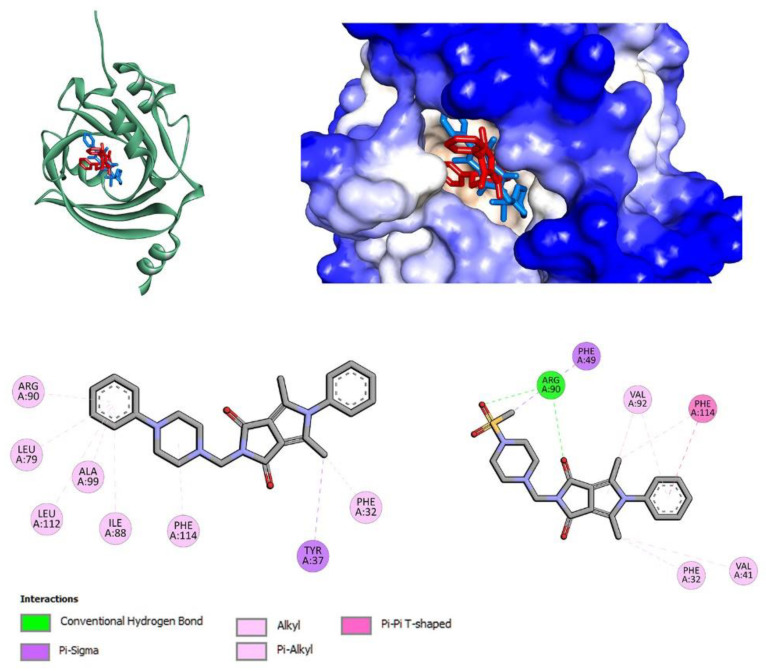
The docked pose of **7a** (red), **7l** (blue) into the pocket of alpha-1-acid glycoprotein (AAG) (IIA–IIB) and 2D interaction plot (**7a**—**left**, **7l**—**right**).

Molecules **7a** (series **a**–**c**) and **7l** (series **l**–**n**) show the strongest interactions with AAG. Figure 13 present the interactions and positions of compounds **7a** and **7l** in the binding pocket of AAG. Several hydrophobic interactions are observed with the 5-phenylpyrrolo[3,4-*c*]pyrrole moiety. For compound **7a**, with the phenylpiperazine group, π–alkyl contacts are observed. For compound **7l**, with methylsulfonyl substituent, a hydrogen bond with Arg90 is formed. The second hydrogen bond is associated between Arg90 and the oxygen atom of the pyrrolo[3,4-*c*]pyrrole group.

### 3.5. In Silico ADME, Physicochemical and Drug-Likeness Predictions

We predicted the Mannich base derivatives **7a**–**7c** and **7l**–**7m** for their possible absorption, distribution, metabolism and excretion (ADME) and physicochemical and drug-likeness properties using the SWISSADME server (http://www.swissadme.ch, date of access: 20 February 2023). Table 16 shows the results describing the physicochemical features, with reference to Lipinski’s rule of five (Ro5) [36]. All studied compounds fulfill the conditions of the Ro5, which means that they will probably show good oral bioavailability and biological membrane permeability. These findings support the data presented in Table 17. Every investigated derivative is supposed to be highly absorbed through the GI tract. Compounds **7a**–**7c** (those with phenylpiperazine substituent) will probably be able to cross the blood–brain barrier (BBB). The predicted water solubility of the derivatives is highly varied (Table 17).

The results of drug-likeness predictions are presented in Table 18. Mannich bases **7a**–**7c** and **7l**–**7m**, apart from fulfilling the Ro5, also meet the descriptors of Veber’s rule [36,88]. Moreover, their calculated topological polar surface area (TPSA) is significantly below the border value of 140 Å^2^, as per the literature [36,37]. These findings and results of the artificial membrane interaction assay suggest that the compounds can easily cross biological membranes.

## 4. Discussion

The *N*-Mannich base derivatives of pyrrolo[3,4-*c*]pyrrole are promising compounds and revealed satisfactory cyclooxygenase inhibitory activity in in vitro and molecular docking studies described in our previous manuscript [25]. The current paper presents a further biological evaluation of six carefully selected derivatives (**7a**–**7c**, **7l**–**7n**) and describes their interactions with artificial models of cell membranes and plasma proteins.

According to the enzymatic assay, the molecules are potent 15-LOX inhibitors, with inhibitory activity comparable to zileuton. These findings are supported by the molecular docking studies. The compounds bind to the active site of 15-LOX, but contrary to zileuton, they neither chelate the iron ion nor interact with amino acids coordinating Fe^2+^. Their potential inhibitory activity relies not on redox mechanism but rather on competing with a natural substrate in binding to the active site of enzyme [14,15,28,49,50,51,52]. Zileuton is actually the only approved LOX inhibitor. Unfortunately, this drug is characterized by disadvantageous pharmacokinetic features and can cause liver toxicity. Therefore, the development of dual COX–LOX inhibitors could provide an efficient and safe anti-inflammatory agents absent of adverse effects characteristic of both NSAIDs and LOX inhibitors. Additionally, such dual inhibitors do not elevate the risk of respiratory inflammation and bronchoconstriction, which can be caused by the overproduction of LTs [14,15,16]. Derivatives of pyrrolo[3,4-*c*]pyrrole meet this concept.

The compounds showed promising antioxidant activity in experiments performed within cells. Oxidative stress often accompanies inflammation and these coexisting process can potentiate each other. Increased levels of ROS and RNS can lead to overexpression of COX and LOX as well. Furthermore, oxidative stress can lead to chain reactions resulting in DNA damage and lipid and protein oxidation. Therefore, drugs that have the ability to scavenge free radicals may play an important role in alleviating inflammation and could protect lipids and chromatin from oxidation [16,30,31,89,90]. All compounds, **7a**–**7c** and **7l**–7**m**, showed protective properties against oxidative and nitrosative stress. After 24 h incubation with NHDF cell culture, they reduced the levels of ROS and RNS. Moreover, the Mannich bases protected chromatin against damage caused by oxidative stress. According to the fast halo assay, all derivatives statistically significantly reduced the number of DNA strand breaks. Protective properties against lipid peroxidation caused by NO were observed for compounds **7a** and **7b**. These results are in good agreement with many reports describing Mannich bases with significant antioxidant activity [90,91]. We previously examined the influence of Mannich base derivatives of pyrrolo[3,4-*d*]pyridazinone on the expression of different proinflammatory mediators. The structural core of those compounds is very similar to the pyrrolo[3,4-*c*]pyrrole scaffold. The derivatives of pyrrolo[3,4-*d*]pyridazinone showed a dose-dependent ability to reduce the level of tumor necrosis factor α (TNF-α), myeloperoxidase (MPO) and PGE_2_ in experiments carried out in vivo [92,93]. The influence on the level of MPO can be explained by their good antioxidative activity [33]. Therefore, it would be worthwhile to investigate also the effect of Mannich bases of pyrrolo[3,4-*c*]pyrrole on the expression of various proinflammatory mediators in in vivo assays.

The effectiveness and usefulness of drugs depend not only on their activity and mechanism of action but also on their pharmacokinetic properties. Drugs administered orally are absorbed in the GIT before they get into the bloodstream, where they can be bound and transported by plasma proteins [35,36,37,86]. According to the calorimetric studies, all investigated compounds added to artificial models of cell membranes influenced the thermotropic properties of phospholipids in a concentration-dependent manner. In the presence of Mannich base derivatives of pyrrolo[3,4-*c*]pyrrole, the interaction between phospholipids became weaker, which suggests that these molecules can cross the cell membrane. This feature is extremely important considering their intracellularly located molecular targets. All these findings are supported by computational studies. According to the data gained by the use of the SWISSADME server, our compounds are characterized by promising pharmacokinetic features. Due to the favorable values of TPSA and MW, they fulfill Veber’s rule and Lipinski’s rule of five. Their GI absorption will probably be high. Both spectroscopic and molecular docking studies revealed that the derivatives bind to HSA and AAG, which might indicate long life in vivo.

## 5. Conclusions

The current paper presents comprehensive in vitro, in silico, calorimetric, spectroscopic and molecular docking studies describing biological, physicochemical and pharmacokinetic properties of *N*-Mannich base derivatives of pyrrolo[3,4-*c*]pyrrole. According to the results, the compounds act as potent anti-inflammatory agents with pronounced antioxidant activity. Moreover, they influence the thermotropic properties of phospholipids in artificial models of cell membranes. Both the results of the calorimetric and computational studies suggest that the compounds can interact with and cross biological membranes. Their interactions with plasma proteins suggest probable long life in vivo. Summarizing, the derivatives of pyrrolo[3,4-*c*]pyrrole are promising molecules that could play an important role in developing drugs used to treat different inflammatory disorders.

## Figures and Tables

**Table 1:** IC_50_ values calculated for 15-lipoxygenase (15-LOX) enzyme; incubation for 5 min with the tested compounds (mean (SD); *n* = 3); statistical significance of 15-LOX inhibition calculated with post hoc test compared to the control with zileuton (* *p* < 0.05).

Compound	IC_50_ [µM]
**7a**	12.63 (0.06) *
**7b**	10.95 (0.04) *
**7c**	12.47 (0.06) *
**7l**	12.81 (0.03) *
**7m**	12.73 (0.05) *
**7n**	14.07 (0.04) *
**zileuton**	13.41 (0.04)

**Table 2:** Binding affinity (kcal/mol) for interaction compounds **7a**–**7c** and **7l**–**7m** with 15-lipoxygenase (15-LOX).

	15-LOX
**7a**	−9.7
**7b**	−8.6
**7c**	−9.0
**7l**	−8.5
**7m**	−7.3
**7n**	−8.8

**Table 3:** The parameters of 1,2-dimyristoyl-sn-glycero-3-phosphocholine (DMPC) main phase transition: temperature (°C) and peak width at half height for 0.1 compound:DMPC molar ratio.

Compound	Tm (°C)	T_1/2_
**7a**	22.65 +/− 0.17	1.10 +/− 0.12
**7b**	21.90 +/− 0.18	1.63 +/− 0.10
**7c**	22.40 +/− 0.08	1.20 +/− 0.12
**7l**	21.73 +/− 0.38	1.55 +/− 0.29
**7m**	22.75 +/− 0.13	0.98 +/− 0.05
**7n**	22.38 +/− 0.15	1.13 +/− 0.15

**Table 4:** ROS scavenging activity of compounds [mean (SD); *n* = 5]; the results were compared to the control and expressed as E/E0 ratios; statistical significance calculated with post hoc test compared to the control (* *p* < 0,05; E0—cultures without tested compounds).

Compound	Concentration [μM]	24 h Incubation with Compounds [E/E_0_]			1 h Incubation with Compounds [E/E_0_]		
		With			With		
		H_2_O_2_			H_2_O_2_		
		Mean	SEM	*p*	Mean	SEM	*p*
**H_2_O_2_** (incubated only 1 h)	100	1.675	0.021	*	1.798	0.033	*
**7a**	100	0.566	0.006	*	0.845	0.021	*
	50	0.575	0.008	*	0.853	0.014	*
	10	0.669	0.003	*	0.888	0.012	*
**7b**	100	0.606	0.004	*	0.949	0.006	*
	50	0.614	0.007	*	1.010	0.008	
	10	0.619	0.006	*	1.121	0.009	*
**7c**	100	0.544	0.003	*	0.939	0.004	*
	50	0.551	0.002	*	1.091	0.004	
	10	0.575	0.005	*	1.226	0.034	*
**7l**	100	0.573	0.006	*	0.851	0.011	*
	50	0.561	0.004	*	0.949	0.004	*
	10	0.544	0.008	*	1.029	0.003	
**7m**	100	0.567	0.009	*	1.286	0.087	*
	50	0.557	0.011	*	1.244	0.023	*
	10	0.547	0.005	*	1.112	0.067	*
**7n**	100	0.631	0.009	*	1.418	0.055	*
	50	0.515	0.006	*	1.418	0.023	*
	10	0.528	0.004	*	1.183	0.045	*

**Table 5:** RNS scavenging activity of compounds [mean (SD); *n* = 5]; the results were compared to the control and expressed as E/E0 ratios; statistical significance calculated with post hoc test compared to the control (* *p* < 0,05; E0—cultures without tested compounds).

Compound	Concentration [μM]	24 h Incubation with Compounds [E/E_0_]			1 h Incubation with Compounds [E/E_0_]		
		With			With		
		H_2_O_2_			H_2_O_2_		
		Mean	SEM	*p*	Mean	SEM	*p*
**SIN-1** (incubated only 1 h)	100	1.982	0.018	*	1.765	0.024	*
**7a**	100	0.845	0.016	*	1.066	0.011	*
	50	0.883	0.026	*	1.129	0.021	
	10	0.922	0.018	*	1.169	0.013	
**7b**	100	0.872	0.020	*	1.071	0.015	
	50	0.832	0.038	*	1.114	0.033	
	10	0.832	0.028	*	1.229	0.023	*
**7c**	100	0.851	0.016	*	0.978	0.011	
	50	0.840	0.010	*	0.964	0.005	
	10	0.834	0.010	*	0.906	0.005	
**7l**	100	0.977	0.014		0.995	0.009	
	50	0.902	0.012	*	0.931	0.004	
	10	0.842	0.039	*	0.853	0.022	
**7m**	100	0.912	0.013	*	0.923	0.011	
	50	0.844	0.012	*	0.855	0.005	
	10	0.838	0.014	*	0.849	0.005	
**7n**	100	0.871	0.017	*	0.881	0.009	
	50	0.802	0.008	*	0.813	0.007	
	10	0.790	0.008	*	0.801	0.034	

**Table 6:** DNA damage evaluation after incubation with tested compounds [mean (SD); *n* = 3]; the results were compared to the control and expressed as E/E0 ratios; statistical significance calculated with post hoc test compared to the control (* *p* < 0,05; E0—cultures without tested compounds).

Compound	Concentration [μM]	24 h Incubation with Compounds [E/E_0_]			1 h Incubation with Compounds [E/E_0_]		
		With H_2_O_2_			With H_2_O_2_		
		Mean	SEM	*p*	Mean	SEM	*p*
**H_2_O_2_** (incubated only 1 h)	100	1.542	0.043	*	1.432	0.053	*
**7a**	100	0.578	0.012	*	0.951	0.021	
	50	0.581	0.022	*	0.958	0.011	
	10	0.599	0.031	*	0.967	0.012	
**7b**	100	0.545	0.041	*	0.911	0.021	
	50	0.580	0.022	*	0.935	0.023	
	10	0.595	0.027	*	0.945	0.021	
**7c**	100	0.554	0.028	*	0.974	0.024	
	50	0.561	0.012	*	1.002	0.021	
	10	0.571	0.015	*	1.054	0.041	
**7l**	100	0.603	0.022	*	1.065	0.027	
	50	0.589	0.027	*	1.024	0.024	
	10	0.528	0.043	*	0.824	0.018	*
**7m**	100	0.642	0.065	*	1.373	0.014	*
	50	0.561	0.054	*	1.324	0.024	*
	10	0.501	0.061	*	1.209	0.034	*
**7n**	100	0.822	0.042	*	1.302	0.024	*
	50	0.565	0.044	*	1.246	0.031	*
	10	0.550	0.057	*	1.045	0.033	

**Table 7:** Lipid peroxidation after incubation with tested compounds [mean (SD); *n* = 3]; the results were compared to the control and expressed as E/E0 ratios; statistical significance calculated with post hoc test compared to the control (* *p* < 0,05; E0—cultures without tested compounds).

Compound	Concentration [μM]	24 h Incubation with Compounds [E/E_0_]			1 h Incubation with Compounds [E/E_0_]		
		With			With		
		H_2_O_2_			H_2_O_2_		
		Mean	SEM	*p*	Mean	SEM	*p*
**SIN-1** (incubated only 1 h)	100	1.987	0.110	*	2.021	0.51	*
**7a**	100	0.827	0.005	*	0.815	0.007	*
	50	0.843	0.007	*	0.838	0.009	*
	10	0.887	0.008	*	0.898	0.010	*
**7b**	100	0.982	0.044	*	0.954	0.061	*
	50	0.877	0.007	*	0.849	0.024	*
	10	0.852	0.009	*	0.824	0.026	*
**7c**	100	1.042	0.014		1.014	0.031	
	50	1.049	0.026		1.019	0.026	
	10	1.039	0.024		1.012	0.031	
**7l**	100	1.085	0.009		1.043	0.016	
	50	1.054	0.019		1.026	0.026	
	10	0.992	0.019		1.019	0.026	
**7m**	100	1.047	0.029		1.093	0.036	
	50	1.054	0.006		1.046	0.013	
	10	1.033	0.005		1.033	0.012	
**7n**	100	1.180	0.007	*	1.048	0.017	
	50	1.040	0.022		1.040	0.032	
	10	1.033	0.030		1.031	0.04	

**Table 8:** The Stern–Volmer constant K_sv_, quenching rate constant k_q_, binding constant K_b_, number of binding sites n, and thermodynamic parameters for the interaction of human serum albumin (HSA) with the compounds at different temperatures.

		Quenching		Binding			Thermodynamic		
	T [K]	K_sv_ × 10^5^ [dm^3^·mol^−1^]	k_q_ × 10^13^ [dm^3^·mol^−1^·s^−1^]	logK_b_	K_b_ × 10^3^ [dm^3^·mol^−1^]	n	ΔG° [kJmol^−1^]	ΔH° [kJmol^−1^]	ΔS° [Jmol^−1^ K^−1^]
**7a**	297 303 308	1.60 ± 0.10 1.23 ± 0.18 0.63 ± 0.06	1.60 1.23 0.63	3.98 ± 0.09 2.93 ± 0.21 2.44 ± 0.30	9.12 0.85 0.27	0.79 ± 0.02 0.65 ± 0.05 0.61 ± 0.07	−22.22	−245.67	−752.33
**7b**	297 303 308	1.36 ± 0.18 1.13 ± 0.13 0.99 ± 0.14	1.36 1.13 0.99	3.66 ± 0.19 2.91 ± 0.22 2.49 ± 0.23	4.57 0.79 0.31	0.73 ± 0.05 0.62 ± 0.04 0.56 ± 0.03	−20.62	−187.54	−562.03
**7c**	297 303 308	1.63 ± 0.13 1.58 ± 0.10 1.33 ± 0.11	1.63 1.58 1.33	3.99 ± 0.13 3.69 ± 0.14 3.18 ± 0.14	9.77 4.90 1.51	0.78 ± 0.04 0.74 ± 0.02 0.66 ± 0.02	−22.95	−127.23	−351.10
**7l**	297 303 308	1.73 ± 0.10 1.09 ± 0.09 0.77 ± 0.08	1.73 1.09 0.77	3.22 ± 0.19 2.90 ± 0.21 2.54 ± 0.22	1.66 0.79 0.35	0.82 ± 0.03 0.62 ± 0.07 0.79 ± 0.04	−18.41	−109.07	−295.15
**7m**	297 303 308	0.29 ± 0.02 0.19 ± 0.01 0.09 ± 0.01	0.29 0.19 0.09	3.80 ± 0.17 3.07 ± 0.30 2.47 ± 0.32	6.31 1.18 0.30	0.88 ± 0.03 0.79 ± 0.05 0.75 ± 0.08	−21.63	−211.64	−639.76
**7n**	297 303 308	0.21 ± 0.01 0.18 ± 0.02 0.11 ± 0.01	0.21 0.18 0.11	3.75 ± 0.23 3.00 ± 0.24 2.35 ± 0.19	5.62 1.12 0.22	0.91 ± 0.05 0.78 ± 0.07 0.70 ± 0.04	−21.47	−222.00	−675.18

**Table 9:** The Stern–Volmer constant K_sv_, quenching rate constant k_q_, binding constants K_b_, number of binding sites n, and thermodynamic parameters for the interaction of alpha-1-acid glycoprotein (AAG) with the compounds at different temperatures.

		Quenching		Binding			Thermodynamic		
	T [K]	K_sv_ × 10^4^ [dm^3^·mol^−1^]	k_q_ × 10^12^ [dm^3^·mol^−1^·s^−1^]	logK_b_	K_b_ × 10^3^ [dm^3^·mol^−1^]	n	ΔG° [kJmol^−1^]	ΔH° [kJmol^−1^]	ΔS° [Jmol^−1^ K^−1^]
**7a**	297 303 308	4.08 ± 0.20 3.44 ± 0.23 2.97 ± 0.17	4.08 3.44 2.97	3.89 ± 0.24 3.24 ± 0.20 2.73 ± 0.28	7.76 1.74 0.53	0.87 ± 0.04 0.77 ± 0.04 0.69 ± 0.05	−22.18	−186.27	−552.71
**7b**	297 303 308	3.08 ± 0.12 2.13 ± 0.17 1.77 ± 0.24	3.08 2.13 1.77	3.41 ± 0.21 2.65 ± 0.25 2.00 ± 0.32	1.57 0.45 0.10	0.81 ± 0.04 0.71 ± 0.04 0.61 ± 0.05	−19.44	−224.21	−689.48
**7c**	297 303 308	4.52 ± 0.31 4.19 ± 0.33 3.25 ± 0.39	4.52 4.19 3.25	3.39 ± 0.18 2.65 ± 0.21 2.31 ± 0.30	9.77 4.90 1.51	0.76 ± 0.03 0.65 ± 0.04 0.60 ± 0.07	−19.05	−175.20	−525.78
**7l**	297 303 308	2.50 ± 0.05 2.06 ± 0.10 1.16 ± 0.18	2.50 2.06 1.16	3.93 ± 0.18 3.31 ± 0.09 2.44 ± 0.31	8.32 2.04 0.28	0.92 ± 0.03 0.82 ± 0.02 0.69 ± 0.06	−20.18	−233.19	−717.16
**7m**	297 303 308	1.69 ± 0.10 1.45 ± 0.17 0.96 ± 0.11	1.69 1.45 0.96	3.42 ± 0.20 2.37 ± 0.29 2.00 ± 0.23	2.63 0.23 0.10	0.86 ± 0.03 0.68 ± 0.05 0.65 ± 0.04	−19.00	−229.09	−707.36
**7n**	297 303 308	1.83 ± 0.10 1.67 ± 0.09 1.40 ± 0.08	1.83 1.67 1.40	3.33 ± 0.23 3.06 ± 0.24 2.76 ± 0.21	2.14 1.15 0.56	0.84 ± 0.04 0.80 ± 0.04 0.75 ± 0.03	−19.02	−91.74	−244.84

**Table 10:** The binding constant of the studied compounds with human serum albumin (HSA) in the presence of site markers phenylbutazone (PHB) and ibuprofen (IBP) at 297 K.

Site Marker	LogK_b_					
	**7a**	**7b**	**7c**	**7l**	**7m**	**7n**
-	3.98 ± 0.09	3.66 ± 0.19	3.99 ± 0.13	3.22 ± 0.19	3.80 ± 0.17	3.75 ± 0.23
HSA+PHB (site I)	3.00 ± 0.22	2.47 ± 0.20	3.20 ± 0.47	2.49 ± 0.17	3.07 ± 0.05	2.90 ± 0.40
HSA+IBP (site II)	2.64 ± 0.07	2.71 ± 0.32	3.05 ± 0.36	2.13 ± 0.30	3.19 ± 0.16	2.51 ± 0.27

**Table 11:** Percentage of content of α-helix in human serum albumin (HSA) in the absence and presence of compounds **7a**–**7c** and **7l**–**7n**, calculated in the CD Multivariate SSE program.

HSA: Analyzed Compound Molar Ratio	**7a**	**7b**	**7c**	**7l**	**7m**	**7n**
**1:0**	69.1%	67.2%	68.0%	67.6%	67.7%	67.6%
**1:0.5**	68.5%	66.7%	67.2%	67.3%	67.0%	66.9%
**1:1**	68.7%	66.4%	66.9%	66.6%	66.7%	66.4%
**1:2**	68.1%	66.3%	66.6%	66.3%	66.5%	66.5%
**1:5**	68.0%	65.8%	66.8%	66.4%	66.2%	66.3%

**Table 12:** Percentage of content of dominating secondary structure elements α-helix and β-sheet in alpha-1-acid glycoprotein (AAG) in the absence and presence of compounds **7a**–**7c** and **7l**–**7n**, calculated in the CD Multivariate SSE program.

AAG: Analyzed Compound Molar Ratio	% α-Helix	% β-Sheet	% α-Helix	% β-Sheet
	**7a**		**7b**	
**1:0**	24.3%	35.3%	22.8%	35.1%
**1:0.5**	24.0%	34.6%	22.9%	35.8%
**1:1**	24.0%	35.8%	22.4%	36.0%
**1:2**	23.8%	35.6%	22.0%	35.8%
**1:5**	23.7%	35.9%	21.9%	36.1%
	**7c**		**7l**	
**1:0**	22.0%	35.9%	21.7%	36.6%
**1:0.5**	21.6%	35.7%	21.4%	36.0%
**1:1**	21.9%	36.9%	21.4%	36.5%
**1:2**	21.9%	36.8%	21.3%	36.4%
**1:5**	21.3%	36.2%	21.5%	37.1%
	**7m**		**7n**	
**1:0**	21.5%	36.4%	21.1%	36.2%
**1:0.5**	21.2%	36.5%	21.4%	37.1%
**1:1**	20.9%	35.6%	20.6%	36.0%
**1:2**	20.9%	36.6%	20.6%	36.9%
**1:5**	20.9%	37.0%	21.2%	36.9%

**Table 13:** Percentage of the secondary structure of free human serum albumin (HSA) and complexes with studied compounds.

Amide I Ingredient Related to Structure	Free HSA	Complex HSA with Compound					
		**7a**	**7b**	**7c**	**7l**	**7m**	**7n**
**β-sheet** (1610–1640 cm^−1^)	25.42	29.47	25.75	26.39	26.42	28.18	25.69
**random coil** (1640–1650 cm^−1^)	3.42	2.35	7.24	8.07	7.69	6.04	6.84
**α-helix** (1650–1665 cm^−1^)	64.55	56.83	56.41	56.53	54.09	59.77	59.64
**β-turn** (1666–1673 cm^−1^)	6.50	10.75	10.05	8.71	11.69	5.53	7.11
**β-antiparallel** (1675–1695 cm^−1^)	0.11	0.60	0.55	0.30	0.11	0.48	0.72

**Table 14:** Percentage of the secondary structure of free alpha-1-acid glycoprotein (AAG) and complexes with studied compounds.

Amide I Ingredient Related to Structure	Free AAG	Complex AAG with Compound					
		**7a**	**7b**	**7c**	**7l**	**7m**	**7n**
**β-sheet** (1610–1640 cm^−1^)	40.21	33.28	36.88	32.89	30.55	34.85	28.73
**random coil** (1640–1650 cm^−1^)	15.33	20.28	18.62	25.63	26.13	23.57	31.41
**α-helix** (1650–1665 cm^−1^)	25.02	30.06	26.95	25.09	26.63	25.80	25.92
**β-turn** (1666–1673 cm^−1^)	9.64	11.40	9.20	11.74	12.51	8.12	11.40
**β-antiparallel** (1675–1695 cm^−1^)	9.79	3.99	8.37	4.66	4.19	7.67	2.54

**Table 15:** The binding affinity (kcal/mol) for interaction compounds **7a**–**7c** and **7l**–**7m** with human serum albumin (HSA) and alpha-1-acid glycoprotein (AAG).

Compound	HSA			AAG
	IIA (PHB)	IIA-IIB (IBU)	IIIA (IBU)	
**7a**	−6.7	−8.9	−5.6	−9.7
**7b**	−8.4	−8.2	−6.0	−8.1
**7c**	−5.6	−8.9	−1.9	−9.2
**7l**	−6.9	−7.6	−7.3	−8.6
**7m**	−8.2	−7.4	−6.3	−8.3
**7n**	−7.7	−8.3	−7.1	−8.5

**Table 16:** Calculated physicochemical properties of investigated derivatives in relation to Lipinski’s rule of five (Ro5) (according to SWISSADME server).

Compound	Physicochemical Properties—Lipinski’s Rule of Five (Ro5)				
	#*H*-Bond Acceptors	#*H*-Bond Donors	Log *P_o/w_* (MLOGP)	MW [g/mol]	#Violations
**7a**	3	0	3.38	414.50	0
**7b**	3	0	2.91	394.51	0
**7c**	3	0	3.85	448.94	0
**7l**	6	0	1.33	416.49	0
**7m**	6	0	0.82	396.50	0
**7n**	6	0	1.82	450.94	0

**Table 17:** Some ADME parameters of investigated derivatives (according to SWISSADME server).

Compound	Pharmacokinetics			
	GI Absorption	BBB Permeability	P-gp Substrate	Water Solubility
**7a**	High	Yes	Yes	Poorly soluble
**7b**	High	Yes	No	Moderately soluble
**7c**	High	Yes	No	Poorly soluble
**7l**	High	No	No	Moderately soluble
**7m**	High	No	No	Soluble
**7n**	High	No	No	Moderately soluble

**Table 18:** Predicted drug-likeness features of investigated derivatives (according to SWISSADME server).

Compound	Drug-Likeness			
	Lipinski	Veber	Bioavailability Score	TPSA [Å^2^]
**7a**	Yes, 0 violation	Yes	0.55	50.48
**7b**	Yes, 0 violation	Yes	0.55	50.48
**7c**	Yes, 0 violation	Yes	0.55	50.48
**7l**	Yes, 0 violation	Yes	0.55	93.00
**7m**	Yes, 0 violation	Yes	0.55	93.00
**7n**	Yes, 0 violation	Yes	0.55	93.00

## Data Availability

Calculations have been carried out in Wroclaw Centre for Networking and Supercomputing (http://www.wcss.wroc.pl, accessed on 20 June 2021).
